# Feasibility Study of a Novel App‐Based Anxiety Intervention for Autistic People

**DOI:** 10.1002/aur.70153

**Published:** 2025-12-14

**Authors:** Bethany Oakley, Charlotte A. Boatman, Saffron Baldoza, Amy Hearn, Colin Larkworthy, Rachel Kent, Ann Ozsivadjian, Sophie Doswell, Antonia Dittner, Amanda Roestorf, Dhara Rawal, Ben Carter, Emily Simonoff, The Molehill Mountain Advisory Group, Saffron Baldoza, Amy Hearn, Colin Larkworthy, Adrian Judd, Marianne Savage

**Affiliations:** ^1^ Department of Forensic and Neurodevelopmental Sciences Institute of Psychiatry, Psychology and Neuroscience, King's College London, Camberwell London UK; ^2^ Molehill Mountain Advisory Group London UK; ^3^ South London and Maudsley NHS Foundation Trust London UK; ^4^ National Adult ADHD and ASD Psychology Service (NAAAPS), Behavioural & Developmental Psychiatry, Monks Orchard House Bethlem Royal Hospital Kent UK; ^5^ Autistica, Suite B London UK; ^6^ Department of Biostatistics and Health Informatics Institute of Psychiatry, Psychology and Neuroscience, King's College London London UK; ^7^ Department of Child and Adolescent Psychiatry Institute of Psychiatry, Psychology and Neuroscience, Camberwell London UK

**Keywords:** anxiety, autism, CBT, digital tools, intervention, mental health, mHealth

## Abstract

At least 50% of autistic people experience clinically relevant anxiety symptoms. However, reasons for elevated rates of anxiety in autism remain poorly understood and there is a high unmet need for novel and adapted therapies for anxiety that are accessible to autistic people. This study aimed to establish the feasibility of a novel app‐based anxiety management tool (“Molehill Mountain”) that has been developed with, and adapted for, autistic people. A single‐centre, single‐arm feasibility study design was employed, whereby autistic people (≥ 16 years) with mild‐to‐severe symptoms of anxiety were recruited to a 13‐week intervention period (King's College London, UK; clinicaltrials.gov identifier NCT05302167). Of 123 prospective participants screened, 100 (81%) participants aged 16–74 years (*n* = 69 female) were enrolled within approximately 15 months. *n* = 76 (76%) completed an anxiety measure at ~15 weeks (Generalized Anxiety Disorder—7 Item Scale; GAD‐7). Most adhered to the full intervention duration: 65% (*n* = 47), with most using the app weekly (1–6 days per week; 58%). 73% of participants agreed that they found the app easy to use overall and that an app is a good format for offering anxiety support to autistic people. There was a significant reduction in self‐reported anxiety symptom severity with mean difference 2.88 (95% CI 1.88, 3.89; *p* < 0.001; Cohen's *d* = 0.45). We found that an autism‐adapted app‐based anxiety management tool is acceptable to the community and associated with reduced anxiety symptom severity in autistic adults, on average. Following optimization to further enhance usability, the efficacy of the Molehill Mountain app for reducing anxiety must now be tested under randomized controlled conditions in a full‐scale clinical trial.

## Summary

At least 50% of autistic people experience anxiety. There are very few effective support options currently available to autistic people to help them to manage anxiety. Autistic people can also experience more barriers to accessing mental health services, as compared to their non‐autistic peers. To address these issues, we tested whether a digital app‐based anxiety intervention (“Molehill Mountain”) could be relevant and useable for autistic adults aged 16‐years and above. We found that the app was possible to implement, acceptable to participants, and was related to reductions in anxiety symptom severity in some autistic adults. We also identified areas for improvement to the app in future.

## 1 Introduction

### 1.1 Background

Anxiety disorders are the most common mental health disorders globally, impacting at least 4% of the world's population (World Health Organization 2023). It is increasingly recognized that clinically relevant anxiety symptoms are over‐represented in neurodivergent populations [e.g., autistic people and those with attention‐deficit hyperactivity disorder; (Lai et al. 2019)]. For instance, up to half of autistic people are estimated to receive a diagnosis of an anxiety disorder by adulthood (Hollocks et al. 2019).

Co‐occurring anxiety in autism has a wide‐ranging impact on the quality of life of individuals and families, which is connected to wider social factors, such as peer relationships, opportunities for engagement in education and employment, and unmet support needs (Adams and Emerson 2021; Cadman et al. 2012; Oakley et al. 2020; Robertson et al. 2018; van Steensel et al. 2012). Thus, providing effective interventions for anxiety management to autistic people is essential, however there are very few existing support options available, and their current evidence‐base is limited (Benevides et al. 2020).

### 1.2 Adapted Anxiety Interventions for Autistic People

The limited availability of effective, evidence‐based anxiety interventions for autistic people may be partly due to differences between autistic and non‐autistic people in the mechanistic underpinnings of anxiety symptoms that represent key targets for support. For example, “atypical” anxiety symptoms (in combination with more traditional presentations of anxiety, as per DSM‐5; (American Psychiatric Association (APA) 2013); also see (Montazeri et al. 2019)) are common in autism, and seem to be linked to the autistic phenotype. For instance, sensory processing differences are experienced at very high rates in autism (APA 2013; Laura Crane et al. 2009) and autistic people and their family members are more likely than their non‐autistic counterparts to report specific phobias linked to aversive sensory stimuli [e.g., vacuum cleaners, facial hair, toilets, mechanical toys; (Kerns et al. 2014; Mayes et al. 2013)]. Additionally, restricted and repetitive behaviors and interests (RRB) are a diagnostic feature of autism and excessive worry about novelty and change, intolerance of uncertainty, and distressed ritualistic and compulsive behaviors not clearly linked to intrusive thoughts or to avoid a dreaded unwanted event (as in obsessive‐compulsive disorder) are further examples of atypical anxiety presentation in autistic populations (Ambrose et al. 2020; den Houting et al. 2019; South and Rodgers 2017).

Potential differences in the etiology and trajectories of anxiety between autistic and non‐autistic populations suggest that adaptations are required when applying anxiety interventions developed with reference to the general population to autistic individuals. Indeed, though Cognitive Behavioral Therapy (CBT) has consistently been shown to be an effective approach for anxiety management in autism (Kreslins et al. 2015; Spain et al. 2015; Sukhodolsky et al. 2013; Ung et al. 2015), a randomized clinical trial with autistic young people demonstrated that autism‐adapted CBT had stronger efficacy than standard of practice CBT (Wood et al. 2020).

Autism‐adapted CBT programmes include strategies, such as highly structured sessions with the use of concrete examples in favor of excessive metaphor and hypotheticals [particularly around identifying and describing emotions that can be more challenging for some autistic people; (Oakley et al. 2020)], allowing for more processing time through regular breaks and the use of visual reminders/other communication aids, and incorporating autistic strengths like personal interests into the therapy to promote continued engagement (Cooper et al. 2018; Spain and Happé 2020; Walters et al. 2016).

### 1.3 Accessible Anxiety Interventions for Autistic People

Although autism‐adapted CBT, alongside other anxiety management strategies that are supported by emerging evidence bases [e.g., mindfulness‐based approaches; (Sizoo and Kuiper 2017; Spek et al. 2013)] are available, many autistic people face additional barriers in accessing these interventions (Brede et al. 2022). For example, autistic people have reported feeling misunderstood by healthcare providers, which can underpin mistrust in the system and subsequent reluctance of individuals to disclose their mental health problems to access the support they need (Brede et al. 2022; Crane et al. 2018). Feelings of being misunderstood can result from a range of factors, including a lack of clinician awareness and understanding of autism, and the healthcare infrastructure being built around neurotypical norms [e.g., in terms of expected communication styles that do not account for autistic needs, like sensory sensitivities and increased preference for predictability and routine; (Doherty et al. 2022; Mason et al. 2019; Nicolaidis et al. 2015; Shaw et al. 2023)]. At the organizational level, this is further linked with the substantial lack of availability of neurodevelopmental specialist pathways within mental health services (National Autistic Society 2019). This means long waiting lists and a high threshold for anxiety symptom severity being required for referral to services, with many autistic people only able to access support through primary care (i.e., maintained contact with GP).

To address some of these challenges, there is increasing interest and investment in the potential of novel digital approaches to complement the delivery of mental health support and/or provide a low‐intensity option before services are required. The potential of digital approaches may be particularly important to increase accessibility for those who experience additional challenges accessing in‐person services [e.g., due to cost, mobility, lack of specialist mental health services in that geographical area; as emphasized by the global COVID‐19 pandemic; (Oakley, Tillman, et al. 2021)]. However, in the context of autism, there is currently very limited evidence for the acceptability and effectiveness of digital technologies implemented to promote better mental health outcomes (Sutherland et al. 2018). From the available evidence, there is an indication that, for those who are able to effectively engage with them [please also see (Noel and Ellison 2020)], digital tools/approaches can offer a low‐intensity, cost‐effective method of providing mental health support to some autistic people (Gaigg et al. 2020). For instance, the only existing study (to our knowledge) of an app‐based psychological intervention specifically designed to target anxiety in autistic people (“HARU ASD”; a CBT program available in Korean) demonstrated a significant decline in self‐reported anxiety for those who used the app over 66 days (*N* = 15), as compared to a waitlist control group [also *N* = 15; (Yang and Chung 2022)].

### 1.4 The Current Study

Here, we report the results of a feasibility study of a novel app‐based anxiety intervention designed with and for autistic people (“Molehill Mountain”). Specific objectives were: (1) To establish the acceptability, feasibility, and usability of the Molehill Mountain app in a real‐world context; and (2) To indicate the target population, performance of selected clinical outcome measures, and ideal timing/duration of intervention to inform the design of a future randomized‐controlled trial.

## 2 Method

### 2.1 Study Design and Setting

This study employed a single‐arm feasibility design with autistic people (≥ 16 years) with mild (or greater) symptoms of anxiety, recruited over a 15‐month period. The study took place at a single research site (King's College London) in the United Kingdom and was sponsored by King's College London and South London and Maudsley NHS Foundation Trust. Ethical approvals were granted by Bromley Research Ethics Committee (IRAS 308723; 22/LO/0291) and all participants provided fully informed written consent prior to their involvement in the study. The study was registered in clinicaltrials.gov (identifier NCT05302167) on 31st March 2022. The protocol was published previously (Oakley et al. 2023) and the study was completed in October 2024.

### 2.2 Community Involvement

Study approaches were developed in consultation with the Molehill Mountain Advisory Group, which comprised seven autistic people and family members. The advisory group was involved in measurement selection and development (please see details below), monitoring and providing feedback on study progress, writing of the statistical analysis plan, and reviewing/co‐authoring publications. Community involvement prior to this study (e.g., in app design/development) is reported elsewhere (Developing the Molehill Mountain App | Autistica).

### 2.3 Participants

One hundred participants were enrolled in the study between 21st October 2022 and 6th January 2024. Participants were screened from NHS outpatient neurodevelopmental/mental health services in the UK (South London and Maudsley, East London, Camden and Islington, North East London NHS Foundation Trusts), non‐profit organizations (i.e., autism charities Autistica and National Autistic Society), and existing research databases/mailing lists where participants had consented to be notified about further research (King's College London, University of Cambridge). Briefly, inclusion/exclusion criteria included being aged 16 years and above, having an existing autism diagnosis (confirmed via clinically administered diagnostic instruments in those recruited from outpatient services, and self‐reported in all other cases), and exhibiting mild to severe symptoms of anxiety at screening. Full details on eligibility are provided in Oakley et al. (2023) and Table S1.

### 2.4 Procedure

**FIGURE 1:**
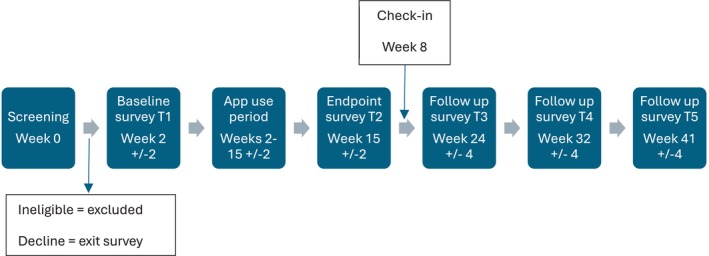
Participant procedures flow diagram.

A study procedures flow diagram is provided in Figure 1. Prospective participants who expressed interest in the study were sent an information sheet and subsequently screened by the research team if they wished to proceed. Those who met eligibility criteria and provided informed consent were sent baseline (Timepoint 1; T1) questionnaires on demographics, clinical outcomes, and assessment of autistic traits (see below).

At the end of the 13‐week intervention period (Timepoint 2; T2), participants were sent an endpoint survey to reassess clinical outcomes and app use experiences. A subsample of participants who consented to this elaborated on their app use experiences via a semi‐structured qualitative interview (full details and qualitative data from interviews are reported elsewhere; Boatman et al. in prep).

Finally, participants were followed up a further three times (Weeks 24, 32, 41 ± 4) to reassess clinical outcomes longitudinally (Timepoints 3, 4, and 5; T3, T4, and T5). All contact between the research team and participants was conducted remotely via the participants' preferred mode of communication (e.g., telephone, video call, live chat, and email). All surveys were administered online via Qualtrics, or the participants' preferred communication method (e.g., pen‐and‐paper, email, and telephone).

Those who declined the study or withdrew were invited to complete an exit survey and/or interview to ascertain reasons for decline or withdrawal.

### 2.5 Intervention

**FIGURE 2:**
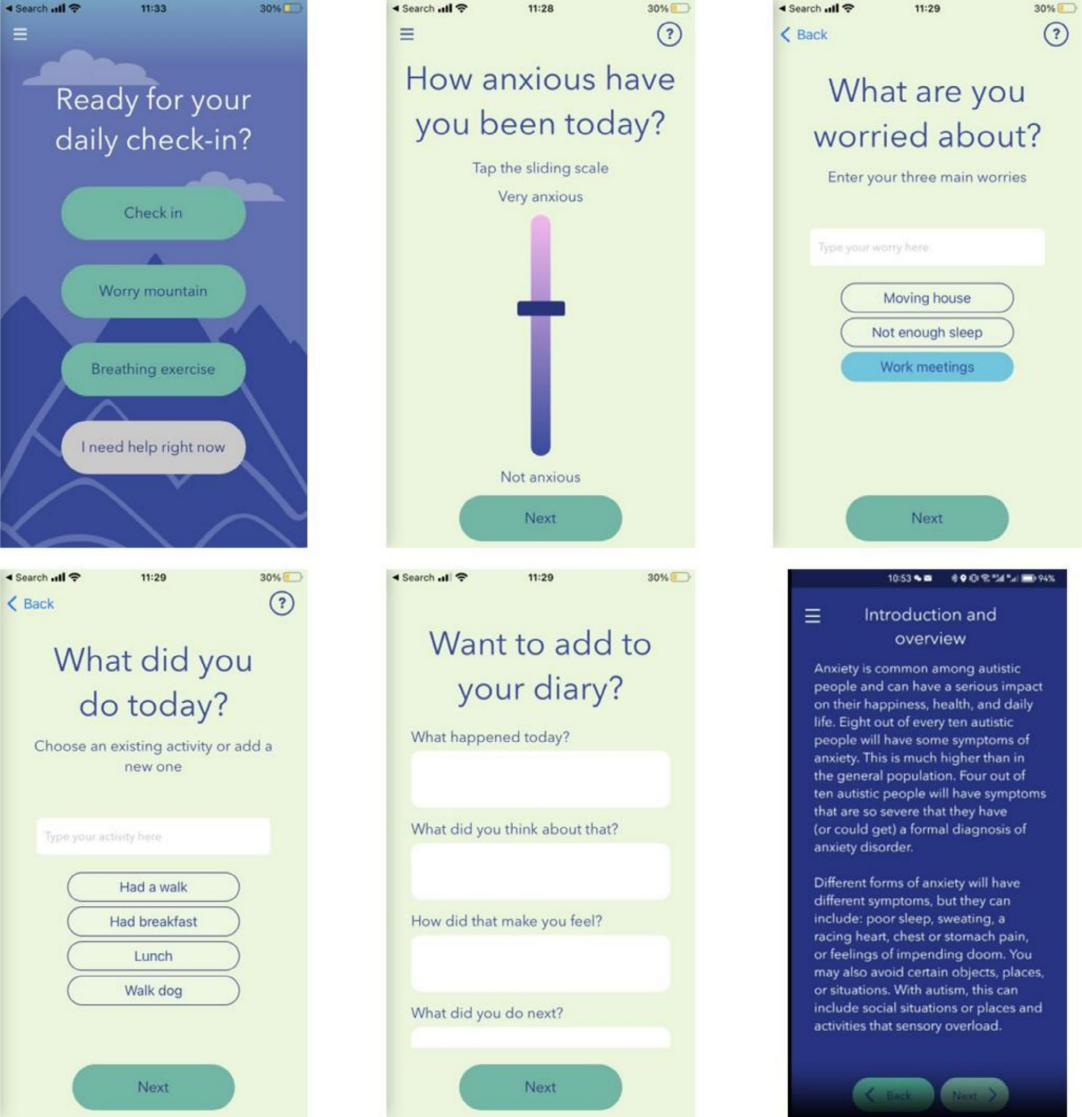
Visuals of the Molehill Mountain app. From top left to bottom right: Home screen; Daily check in (Anxiety Sliding Scale); Daily check in (Worry Entry); Daily check in (Activities Entry); Daily check in (Diary); Daily check in (Tips and Tools unlocked). Republished for illustrative purposes from the Molehill Mountain app V2.6 under a CC BY license, with permission from Autistica and King’s College London, original copyright 2017.

We implemented the freely available public version of the Molehill Mountain app (currently Version 2.6.3), which is compatible with iOS and Android. App technical maintenance and development were provided by EqualEyes (and subsequently Old St Labs). Full details on app development and content are available elsewhere (please see Oakley et al. 2023). Figure 2 visually demonstrates key app features.

Participants were supported by the research team to download and initiate the Molehill Mountain app and were invited to use the app daily for 13 weeks, with a check‐in at 8 weeks to address any concerns or queries that may have arisen.

Briefly, the main active component of the app is the tips and tools section that is progressively unlocked through engagement with the app over 13 weeks. Tips and tools embedded in the app are designed by clinicians (RK, AO, ES) and underpinned by autism‐adapted cognitive behavioral therapy (CBT) principles (e.g., exposure, practical problem solving, and cognitive restructuring), integrating psychoeducation on common autistic experiences of anxiety, with a focus on emotional literacy, perseveration, managing uncertainty and change, generalization of anxiety management strategies across situations and contexts, and sensory and social anxiety. Additional features include daily check‐in to report on worries, anxiety levels and activities; diary function to record daily experiences; progress tracker to monitor personal app usage and anxiety change; low‐intensity anxiety management techniques, including guided breathing and “worry time” (i.e., control over setting aside a short period of time in the day to engage with anxiety) as in‐app interactive features; and signposting for further support.

We asked for participant consent to access objective app usage data, which was agreed to and available for 77 participants and included metrics that enabled us to ascertain “dose response” (i.e., number of check‐ins, progress in unlocking tips and tools).

### 2.6 Materials and Measures

Autistic traits were assessed at T1 only, clinical outcome measures at all timepoints (T1–T5), and app usage experiences at T2 (with brief additional questions regarding any continued use of the Molehill Mountain app included in T3–T5). We note that one participant reported filling in surveys with support from someone else (mother).

#### 2.6.1 App Experience Survey

To address the primary aim of establishing the acceptability/useability and feasibility of the Molehill Mountain app, we included a self‐reported App Use Experience survey (please see Table S2) that was co‐designed with the Molehill Mountain Advisory Group to assess key feasibility indices derived from prior research [(Baumel et al. 2017) e.g., usage/discontinuation patterns, ease of use, technical/security aspects, feature/design preferences]. In this survey, we also assessed participants' perceptions of wider study approaches (e.g., subjective experiences of anxiety over time) and their views on future clinical trials of app‐based anxiety interventions (e.g., willingness to be randomized).

#### 2.6.2 Measurement of Anxiety

The core measure of anxiety symptom severity in this study was the self‐report 7‐item Generalized Anxiety Disorder Assessment [GAD‐7; (Spitzer et al. 2006)]. Items refer to anxiety features in the last 2 weeks, rated on a scale from 0 (Not at all) to 3 (Nearly every day), with higher scores indicating higher anxiety symptom severity and thresholds established for Mild (scores 5–9), Moderate (scores 10–14), and Severe (scores 15–21) anxiety. GAD‐7 is a widely used anxiety assessment tool in UK clinic services and in recent autism trials (Rai et al. 2024; Russell et al. 2017). Its short format can reduce participant time/burden, and it has been recently shown to have good psychometric properties in an autistic college sample (Robeson et al. 2024). In this study, Cronbach's alpha indicated good internal consistency (*α* = 0.82).

#### 2.6.3 Measurement of Additional Clinical Outcomes

The self‐report 14‐item Hospital Anxiety and Depression Scale [HADS; (Zigmond and Snaith 1983)] and individualized functional outcome via Goal Attainment Scaling (GAS; Oakley et al. 2023) was additionally included to assess other clinical outcomes that may be associated with anxiety.

The HADS assesses anxiety/depression features in the past week, rated from 0 to 3 (8 items reverse coded), with higher scores indicating higher anxiety/depression symptom severity and thresholds established for “Borderline” (scores 8–10) and “Clinically Relevant” (scores 11–21) cases.

The GAS, co‐produced with the Molehill Mountain Advisory Group, is a 20‐item questionnaire that assesses a range of personal goals, anchored to anxiety (e.g., “I would like to feel less anxious about different sensory environments or triggers”). The GAS can be viewed in full in S1 Material in S1 file of Oakley et al. (2023). At T1, participants were asked to select a maximum of 4 out of the 20 personal goals that they wished to work toward over the course of the intervention and to rate them according to importance (from 1 Not at all important to 4 Very important) and how far away from achieving that goal they felt right now (from 1 A long way from this goal to 5 I have exceeded this goal by a lot). At T2 and T3–T5, participants were asked to update on how far away they felt from achieving their 4 personal goals.

#### 2.6.4 Autistic Traits

Self‐reported autistic trait measures were selected by the Molehill Mountain Advisory Group for the purposes of T1 characterization only (our intervention target was anxiety change and not change in autistic features). Traits were assessed via the Adult Routines Inventory‐Revised [ARI‐R; (Evans et al. 2017)] and the Comprehensive Autistic Trait Inventory [CAT‐I; (English et al. 2021)]—tools selected by the Molehill Mountain Advisory Group.

The CAT‐I contains 42 items that assess social interactions, communication, social camouflage, repetitive behaviors, and sensory sensitivity. Each item is scored from 1 (Definitely Disagree) to 5 (Definitely Agree) (five items reverse coded), with higher scores representing more autistic traits (possible subscale scores range from 7 to 35; possible total scores range from 42 to 210).

The ARI‐R includes 55 items that reflect restricted and repetitive behaviors across a spectrum, from those seen in neurotypical development (e.g., bedtime rituals), to those frequently associated with neurodivergence (e.g., compulsions, motor stereotypies, tics, sensory sensitivities, resistance to minor changes in routine and environments). Each item is scored from 1 (Not at all/Never) to 5 (Very much/Always; two items reverse coded), and total possible scores range from 55 to 275.

### 2.7 Statistical Analysis

**FIGURE 3:**
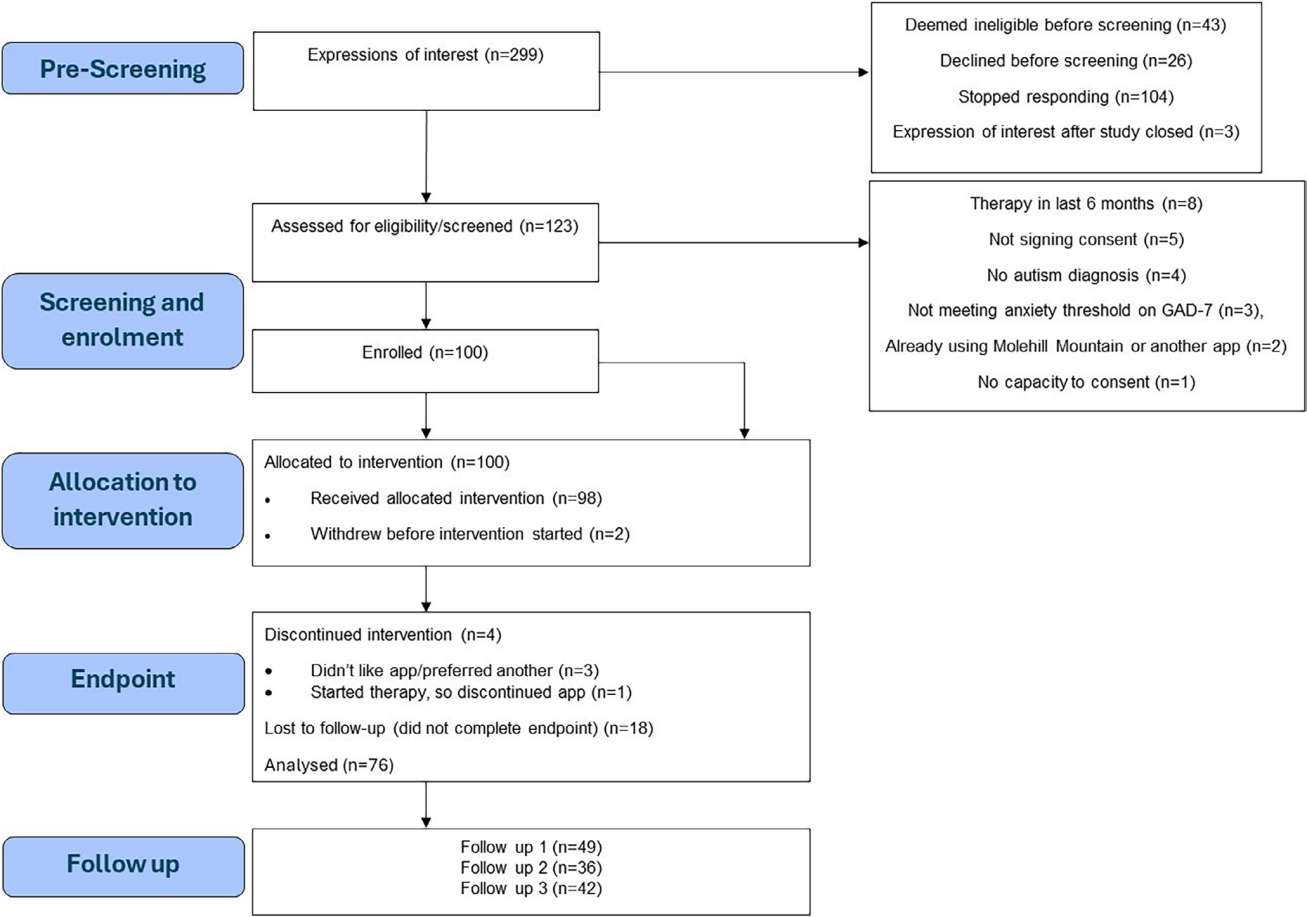
CONSORT flowchart.

The pre‐registered statistical analysis plan for this study is available via Open Science Framework (https://osf.io/8nwek). Results are reported following the Consolidated Standards of Reporting Trials (CONSORT) checklist for transparency, alongside the CONSORT flow chart (Figure 3). Data were analyzed using R Version 4.4.2.

We descriptively summarize participant characteristics, clinical outcomes, and feasibility indices.

Changes in anxiety symptoms (GAD‐7) were assessed using a paired t‐test (screening vs. T2) and mixed‐effects linear regression (screening, T2, T3–T5; “lmerTest” package), with fixed effects for age, sex, autistic traits (CAT‐I Total), app usage frequency (number check‐ins), and timepoint. A random intercept was fitted for each participant and the mean difference (MD) and adjusted MD (aMD) were reported.

For changes on additional clinical outcomes pre‐post intervention, we report paired t‐tests (screening vs. T2). Changes on the GAS are reported descriptively, due to the nature of the scale.

Where < 20% of items from a questionnaire were missing, the missing values were imputed based on the average of other items from that (sub)scale (GAD‐7 *N* = 4 at T2, *N* = 1 at T3, *N* = 1 at T4; HADS *N* = 2 at T1, *N* = 5 at T2, *N* = 2 at T3, *N* = 1 at T4; ARI *N* = 8; CAT‐Q *N* = 10). No completed measures included >20% missing items.

## 3 Results

### 3.1 Participant Recruitment and Retention

A total of 299 prospective participants had interactions with the research team and 123 prospective participants were screened. One hundred (81%) participants enrolled in the study (Figure 3), with *n* = 76 (76%) followed up, based on completion of the GAD‐7 at T2. There were six active withdrawals (two prior to using the app; Figure 3), with one of these due to not liking the app as it increased anxiety. There were no serious adverse events. Attrition characteristics are reported in Table S3.

### 3.2 Participant Characteristics at Baseline

#### 3.2.1 Demographic Characteristics

At T1, participant age ranged from 16 to 74 years, with a mean age of 40.94 years (SD = 14.03; Table 1). Two thirds of participants reported female sex at birth (*n* = 69) and one third male sex at birth (*n* = 30). For gender identity, two participants reported third gender/non‐binary, two participants reported agender/non‐gendered identity (self‐described), and five participants declined to answer.

**TABLE 1:** Participant characteristics at baseline (T1).

		*N*			
Demographics	Sex (Female: Male)^a^	69:30			
	Gender identity				
	Female	59			
	Male	31			
	Third gender/non‐binary	2			
	Other (self‐describe)^b^	2			
	Prefer not to answer	5			
	Ethnicity				
	White	88			
	More than one	4			
	Black	2			
	Asian	1			
	Other (self‐describe)^b^	2			
	Prefer not to answer	2			
	Living arrangements				
	Independently	70			
	Parents/family	21			
	Daily/regular support	4			
	Prefer not to answer	4			
	Education/employment				
	Full‐time	46			
	Part‐time	21			
	Voluntary	7			
	Not in	19			
	Retired or carer	5			
	Co‐occurring diagnoses				
	Anxiety	86			
	Depression	67			
	Specific learning difficulties	24			
	ADHD	21			
	Eating disorders	16			
	OCD	13			
	Tics/Tourette's	4			
	DCD	2			
	Psychosis	2			
	Bipolar	2			
	Conduct problems	1			
	Medications	59			

	Therapy (past 6 months)	12			
		*N*	Mean	Standard deviation	Range
	Age (years)	99	40.94	14.03	16–74
Age of autism dx	99	34.31	15.89	4–69
Autistic traits	CAT‐I (Total)	89	157.47	21.04	79–193
	CAT‐I (Social)	89	26.61	4.71	13–35
	CAT‐I (Communication)	89	24.12	4.60	10–33
	CAT‐I (Camouflage)	89	25.20	4.40	11–33
	CAT‐I (Rigidity)	89	28.08	5.04	11–35
	CAT‐I (RRB)	89	25.79	5.02	11–34
	CAT‐I (Sensory)	89	27.67	6.20	8–35
	ARI‐R (Total)	89	189.91	34.35	80–265
Anxiety (and depression)	GAD‐7 (Total)^c^	100	13.23	4.59	5–21
	HADS (Anxiety)	89	14.70	3.40	6–21
	HADS (Depression)	89	9.39	4.49	0–19

Abbreviations: ADHD = Attention Deficit Hyperactivity Disorder; ARI‐*R* = Adult Routines Inventory—Revised; CAT‐I (RRB) = Comprehensive Autistic Trait Inventory Restricted and Repetitive Behaviors Subscale; CAT‐I = Comprehensive Autistic Trait Inventory; DCD = developmental coordination disorder; dx = diagnosis; GAD‐7 = Generalized Anxiety Disorder—7 Item Scale; HADS = Hospital Anxiety and Depression Scale; *N* = number of participants; OCD = obsessive‐compulsive disorder.

^a^ Represents sex at birth; gender identity is reported in the main text.

^b^ Gender identity: agender (*n* = 1), non‐gendered (*n* = 1); Ethnicity: Jewish (*n* = 1), Irish (*n* = 1).

^c^ Primary anxiety measure (assessed during screening to confirm inclusion criteria).

Most participants were of white ethnicity (*n* = 88), were living independently (*n* = 70), and almost half were currently in full‐time employment or education (*n* = 46).

#### 3.2.2 Clinical Characteristics

The average age of autism diagnosis in the sample was 34 years (range 4–69 years; SD = 15.89). *n* = 86 participants had an existing self‐reported co‐occurring anxiety diagnosis (not clinically confirmed). Additional existing self‐reported co‐occurring diagnoses included Depression (*n* = 67), Specific Learning Problems (e.g., Dyslexia/Dyscalculia; *n* = 24), Attention‐Deficit Hyperactivity Disorder (*n* = 21), Eating Disorders (*n* = 16), Obsessive‐Compulsive Disorder (*n* = 13), Tics/Tourette's (*n* = 4), Developmental Coordination Disorder (*n* = 2), Psychosis/Schizophrenia (*n* = 2), Bipolar (*n* = 2), and Oppositional Defiant/Conduct Disorder (*n* = 1).

A *n* = 59 reported currently using at least one medication (with antidepressants the most common class), and *n* = 12 accessed therapies in the past 6 months (fewer than 6 sessions).

#### 3.2.3 Autistic Traits

The average total score for autistic traits on the CAT‐I in the sample was 157.47 (range 79–193; SD = 21.04). Average CAT‐I subscale scores were relatively comparable (Table 1), with the highest average score for the subscale of cognitive rigidity (*M* = 28.08, SD = 5.04) and the lowest for the subscale of communication (*M* = 24.12, SD = 4.60). On the ARI‐R, the average total score in the sample was 189.91 (range 80–265; SD = 34.35).

#### 3.2.4 Anxiety (and Depression)

At T1, the average GAD‐7 anxiety score in the sample was 13.23 (SD = 4.59), with 20% (*n* = 20) scoring in the “Mild” range, 46% (*n* = 46) in the “Moderate” range, and 34% (*n* = 34) in the “Severe” range for anxiety symptom severity.

On the HADS, the average anxiety score in the sample was 14.70 (range 6–21; SD = 3.40), and the average depression score was 9.39 (range 0–19; SD = 4.49).

#### 3.2.5 Goal Setting

Participant responses on the GAS are reported in full detail in Table S4. The most frequent personal goals that over one‐third of participants selected to work toward over the course of the intervention were: Goal 5 “I would like to feel less anxious about social situations, being out in public, communicating, and talking to others” (*n* = 37; 42% of respondents), and Goal 3 “I would like to feel less anxious about change (e.g., learning new ways to do things, disruptions in routine/plan, when something is out of order or out of place, etc.)” (*n* = 36; 41% of respondents).

Notwithstanding the specific personal goals selected by individual participants, goals were rated as “*Moderately Important*” to participants, who also reported feeling “*A long way*” from achieving them right now, on average.

### 3.3 App Feasibility and Acceptability

Participant responses to the T2 App Use Experience survey are reported in full detail in Table S2.

#### 3.3.1 App Usage Patterns

Most (65%; *n* = 47) respondents self‐reported using the app for the full 13‐week duration (recommended). About 17% (*n* = 12) self‐reported using the app daily (recommended), with most other respondents (56%, *n* = 40) using the app weekly (4–6 days per week *n* = 31, 1–3 days per week *n* = 9). Almost all respondents (99%, *n* = 70) reported a typical duration of ≤ 15 min per app check‐in.

App data demonstrated an average of 101 check‐ins per participant to record worries (range 1–361; SD = 74.88), with participants unlocking an average of 60/80 (range 1–80; SD = 21.42) tips and tools over the course of the study.

The most commonly entered worry type (as categorized by participants) was specific phobia (*n* = 653), followed by general anxiety (*n* = 426), social anxiety (*n* = 237), and sensory anxiety (*n* = 88). *n* = 94 worries entered were not assigned to any worry type by participants (suggesting some difficulties with identification/differentiation of worry types).

#### 3.3.2 Ease of Technical Use

Most respondents (73%, *n* = 52) found the app easy to use overall, and felt that an app is a good format for offering anxiety support to autistic people (73%, *n* = 30), albeit some had concerns over data security and privacy (14%, *n* = 6). Some respondents also experienced technical issues with the app (e.g., issues with loading the app, revisiting where they last left off; 29%, *n* = 21).

#### 3.3.3 App Feature Preferences

Most respondents (83%; *n* = 35) found the visual design of the app appealing. Individual app feature preferences varied; though overall, the daily check‐in function was rated as the “most useful” app feature most frequently (44%, *n* = 14) and the ability to see/track personal progress statistics in the app was rated as “least useful” most frequently (34%, *n* = 11).

#### 3.3.4 Study Design Preferences

In terms of the suitability of the anxiety measures utilized in this study, 85% (*n* = 36) of participants who responded to the question “Were the anxiety questionnaires used in this study appropriate/relevant to your experiences?” agreed “Very Much” (33%; *n* = 14) or “Somewhat” (52%; *n* = 22).

The vast majority (94%; *n* = 66) of respondents would consider taking part in a clinical trial of a mobile app to support autistic people with anxiety in the future, and 93% (*n* = 66) would be willing to be randomized (as long as they could access the app after the trial had finished, regardless of trial arm).

### 3.4 Anxiety Change Pre‐Post Intervention

#### 3.4.1 Anxiety Outcome (GAD‐7)

**FIGURE 4:**
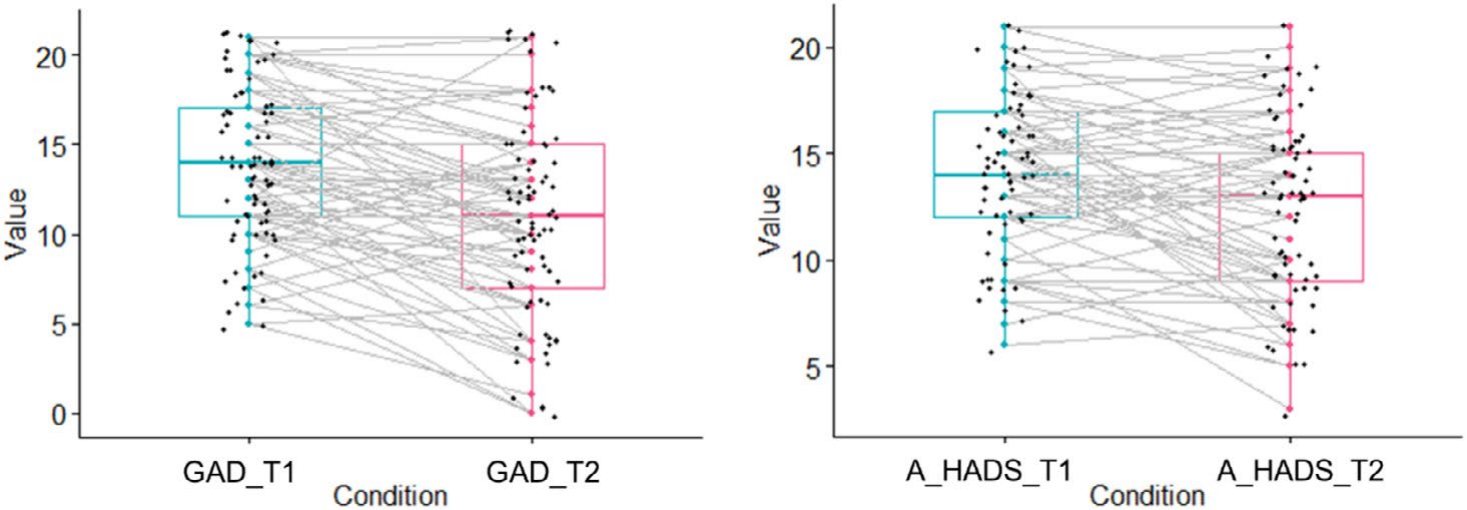
Pre‐post comparison of anxiety scores on the GAD‐7 (left) and HADS Anxiety Subscale (right). GAD_T1 = Generalized Anxiety Disorder—7 item scale score at baseline. GAD_T2 = Generalized Anxiety Disorder—7 item scale score at endpoint. A_HADS_T1 = Anxiety Subscale of Hospital Anxiety and Depression Scale at baseline. A_HADS_T2 = Anxiety Subscale of Hospital Anxiety and Depression Scale at endpoint.

At T2, there was a significant average reduction in self‐reported anxiety on the GAD‐7, as compared to screening: MD = 2.88 (95% CI 1.88, 3.89; *p* < 0.001; Table 2 and Figure 4), with a Cohen's *d* effect size of 0.45.

**TABLE 2:** Participant characteristics on anxiety (and related) measures at T2–T5.

	Endpoint (T2)			Follow up 1 (T3)			Follow up 2 (T4)			Follow up 3 (T5)		
	*N*	Mean (SD)	Range	*N*	Mean (SD)	Range	*N*	Mean (SD)	Range	*N*	Mean (SD)	Range
GAD‐7 (Total)	76	10.96 (5.58)	0–21	49	11.82 (5.69)	0–21	36	11.72 (5.51)	2–21	42	10.62 (5.36)	0–21
HADS (Anxiety)	75	12.49 (4.30)	3–21	48	13.06 (4.43)	2–20	36	13.25 (4.13)	2–21	41	11.63 (4.05)	4–20
HADS (Depression)	75	7.88 (5.41)	0–21	48	9.27 (5.45)	0–21	36	9.14 (5.59)	0–20	41	8.32 (4.86)	0–18

Abbreviations: GAD‐7 = Generalized anxiety disorder – 7 item scale; HADS = Hospital Anxiety and Depression Scale; *N* = number of participants; SD = standard deviation.

**FIGURE 5:**
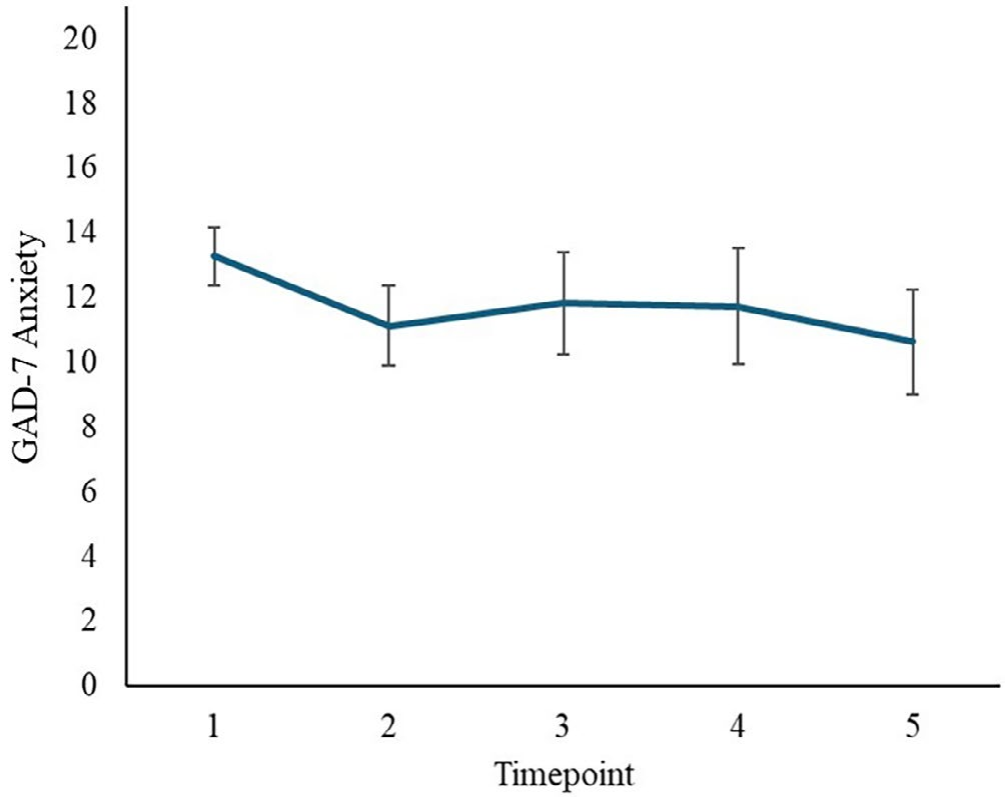
Average anxiety symptom severity self‐reported ratings on the primary outcome measure (Generalized Anxiety Disorder—7 Item Scale; GAD‐7) at each timepoint: 1 = Screening/baseline; 2 = Endpoint; 3 = Follow up 1; 4 = Follow up 2; 5 = Follow up 3.

Modeling of GAD‐7 scores from across all timepoints (screening to T5) showed a significant effect of timepoint (*p* < 0.001). Post hoc analyses indicated a significant reduction in GAD‐7 scores between screening and all other timepoints: T2 (aMD = 2.86, 95% CI [1.25–4.47]; *d* = 0.88, 95% CI [0.51, 1.25]); T3 (aMD = 2.13, 95% CI [0.19, 4.06]; *d* = 0.65, 95% CI [0.22, 1.09]); T4 (aMD = 2.30, 95% CI [0.22, 4.39], *d* = 0.71, 95% CI [0.24, 1.18]); and T5 (aMD = 3.55, 95% CI [1.52, 5.58], *d* = 1.09, 95% CI [0.62, 1.56]); but no further decline in GAD‐7 scores after T2 (*p* ≥ 0.41; see also Figure 5). There was also a significant effect of T1 autistic traits in this model (higher autistic traits associated with higher GAD‐7 scores; *p* < 0.001) and a marginal effect of age (*p =* 0.05), but no significant effect of sex (*p* = 0.57), nor app usage frequency (*p* = 0.10).

#### 3.4.2 Anxiety (HADS and Subjective Report)

There was a similar significant reduction in self‐reported anxiety symptoms on the HADS: MD = 1.68 (95% CI 0.84, 2.52; *d* = 0.58; *p* < 0.001; Figure 4).

These findings were complemented by participants' subjective feedback on the impact of Molehill Mountain on their anxiety, via the App Use Experience survey. Here, 74% (*n* = 52) of respondents felt that the app had helped them to *understand* their anxiety better (by “a little” *n* = 24, or “quite a lot/very much” *n* = 28), and 64% (*n* = 46) felt it had helped them to *manage* their anxiety better (by “a little” *n* = 37, or “quite a lot/very much” *n* = 9).

About 68% (*n* = 27) of respondents felt that the strategies provided by the intervention would support them with anxiety in their daily life going forward, particularly in terms of journalling and engaging in reflection on anxiety.

Figure 4 illustrates individual variation in pre–post anxiety change, with some participants also reporting that the app/features did not help with (or increased) anxiety (*N* = 17; please see Supplementary Table 2).

#### 3.4.3 Additional Clinical Outcomes

There was a significant change in HADS Depression subscale score MD = 1.72 (95% CI 0.73, 2.72; *d* = 0.31; *p* = 0.001).

On the GAS, at T2 participants were most likely to report feeling “A little way” from achieving their selected goals (regardless of the specific goals chosen) on average, representing a 1‐point change.

## 4 Discussion

### 4.1 Molehill Mountain App Feasible and Acceptable to the Community

This study represented the largest effort, to our best knowledge, to test the feasibility of a novel app‐based anxiety management tool (“Molehill Mountain”) that has been developed with, and adapted for, autistic people. We established strong evidence for the feasibility of our approaches against indices of recruitment and retention rate, and intervention adherence (Oakley et al. 2023). We met our original recruitment target of *N* = 100 within approximately 15 months, coordinated from one lead site (King's College London, UK). 81% of participants screened were deemed eligible and consented to enroll, and we achieved a 76% retention rate on our primary outcome measure from screening to endpoint (T2; 81% of these participants also went on to complete at least one optional follow‐up assessment).

Moreover, we found the app to be acceptable to autistic adults. 66% of participants used the app for the full 13‐week duration (recommended), with most using the app weekly (1–6 days per week; 58%), and fewer (16%) using the app daily (recommended). These app usage parameters are relatively in line with (and in some cases stronger than) prior reports [e.g., (Li et al. 2025; Spaargaren et al. 2025; Tromans et al. 2023)], though in the broader mobile health app literature these parameters are often not reported in detail, with specific quality standards for reporting and evaluation now emerging (Ribaut et al. 2024). Comparison across existing studies implementing mobile health apps in autistic adult populations is also challenging due to wide variation in approaches (e.g., intervention type and target, intended delivery duration/frequency, and target population). Though we cannot directly extrapolate uptake and adherence indices from this study to a non‐research context (where app users will have no line of communication with a research team), the freely available public version of Molehill Mountain has been downloaded by 92,300 users since the 2021 launch and currently has over 16,300 active users, also suggesting that if further evidence for efficacy can be provided, this anxiety management tool has the potential for high uptake in a real‐world setting.

In addition to promising data on app usage duration/frequency, most participants agreed that they found the app easy to use overall and that an app is a good format for offering anxiety support to autistic people (73%). Participants indicated that the app‐based format of Molehill Mountain provided easy access to support adapted to autistic needs without the requirement for social contact (or while waiting for/between therapy) and with a structured/visual format that could be integrated into the individuals' everyday routine.

### 4.2 Molehill Mountain App Impact on Anxiety Symptoms

We also established the first evidence for an on average reduction in anxiety symptom severity in autistic adults following the use of the Molehill Mountain app, on two widely used clinical measures (GAD‐7; HADS‐Anxiety), and this was maintained in those who completed follow‐up assessments up to 6 months later. This aligns with recent research demonstrating the impact of a transdiagnostic mental health tool (Brain in Hand) on anxiety reduction in autistic adults over 12 weeks (Tromans et al. 2023), adding further weight to the argument that self‐guided digital tools may be a low cost/scalable support option that is effective for some individuals (Backman et al. 2024; Gaigg et al. 2020)—especially tools that integrate CBT (Grist et al. 2019; Lehtimaki et al. 2021).

As demonstrated by responses to the App Use Experience survey, participants attributed the positive impact of the app primarily to improvement in understanding their own anxiety and triggers better and being better able to describe these to others. Additional benefits indicated by participants after having used the Molehill Mountain app included fewer episodes of “getting stuck” in cycles of repetitive negative thoughts and a decrease in masking of autistic traits. Around one‐third of participants further endorsed that the Molehill Mountain app had directly supported them to manage their anxiety better, and that the strategies they had learned would be useful in the future.

Improved ability to identify and describe anxiety and triggers suggests that app content supported participants with emotion awareness, which is key for implementing adaptive emotion regulation strategies and eliciting social support (Gross 2015; Riedelbauch et al. 2023) and may also reduce more maladaptive coping strategies like masking. Additionally, reduced negative rumination following app use affirms widely reported evidence of the mechanistic action of CBT on cognitive change [central to the theory of CBT; (Lorenzo‐Luaces et al. 2015)], here in the context of an autism‐adapted digital tool. Overall, these findings begin to establish that app content is acting on key targets of emotion awareness difficulties and perseverative thinking that are known to be elevated in autism (APA 2013; Bird and Cook 2013; Gotham et al. 2014; Kinnaird et al. 2019) and associated with increased anxiety and other mental health symptoms in autistic populations (Josyfon et al. 2023; Maisel et al. 2016; Oakley et al. 2020). Nevertheless, we did not include additional measures that would have enabled us to directly test these candidate mechanistic pathways, and this is a key direction for future work.

We also acknowledge the importance of individual differences, as demonstrated by individual data points shown in Figure 4, indicating that while many participants experienced a reduction in anxiety symptom severity post‐intervention, for others anxiety was relatively stable, or increased. This further emphasizes the broader point that no single intervention approach will be appropriate nor effective for all autistic adults. In a future fully powered trial it will be important to consider whether specific subgroups of autistic adults may benefit most from the Molehill Mountain app—for instance, here we reported associations between anxiety change and baseline autistic traits (and, to a lesser extent, age).

### 4.3 App Optimization

Though our findings are promising, another core aim of this feasibility study was to identify areas where improvement to the Molehill Mountain app would be required to maximize usability and beneficial impact for the community. For participants who reported issues with the app/did not perceive it as useful for anxiety management, this was most frequently explained by: (a) needing more frequent reminders (and/or greater control over timing and delivery of reminders to avoid overload) to engage with the app—including on “good days” (that participants identified as important, but where they were less likely to remember to use the app than on “bad days”), and (b) a desire for greater personalization options within the app, including in terms of the range of features available and how participants could interact with them.

In agreement with the broader digital mental health interventions literature, our results also emphasized that technical development and user support (to avoid/quickly and effectively mitigate any app technical issues), in addition to ensuring and communicating strong standards of data privacy and security, are priority areas to ensure uptake and continued engagement (Bevan Jones et al. 2023; Catania et al. 2024).

### 4.4 Strengths

An overarching strength of this study was its multidisciplinary approach, bringing together expertise from academia (study protocol/implementation and statistical approaches), clinical psychology/psychiatry (clinical content of app features, and interpretation), the charity sector (liaison between study team, industry, and user community, access to objective app usage data), industry (app technical development and maintenance), and lived experience. We centered the views and preferences of autistic people on the approaches and measures to be utilized, through our Molehill Mountain Advisory Group. This is likely important in explaining the high endorsement by participants of the study design (e.g., suitability of anxiety measures included) in responses to the App Use Experience survey at endpoint (T2). Co‐design with the Molehill Mountain Advisory Group was also imperative for capturing a wider and richer range of information about participants' intervention goals through the Goal Attainment Scale, and their app use and anxiety experiences through the App Use Experience survey. While more in‐depth qualitative insights from participants were beyond the scope of this paper, we report them elsewhere (Boatman et al. in prep).

### 4.5 Limitations

There are also several limitations that must be considered when interpreting results. Firstly, our pre‐post study design limits our ability to infer the efficacy of the app for reducing anxiety and our findings of reduced self‐reported anxiety symptom severity on the GAD‐7 and HADS can thus only be considered preliminary signals for effectiveness. Indeed, lack of adequate comparator and blinded outcome assessment have been identified as key gaps in the digital mental health interventions field as a whole, with pre‐post designs tending to demonstrate inflated effect sizes for intervention effectiveness (Hollis et al. 2017). Our results did not confirm a dose–response relationship between increasing app usage and anxiety reduction over time, which further emphasizes the need for a randomized‐controlled trial to differentiate the impact of the app, specifically, from the positive/expectancy effects of access to support more generally [i.e., placebo effects; (Patterson et al. 2016)], natural variation in symptoms over time, and regression to the mean. This study supports that such a trial is warranted and represents a critical first step in informing its design and approaches.

Our core clinical outcome measure (GAD‐7) was developed with reference to the general population and thus there may be some differences in anxiety manifestation and item interpretation when applied to autistic populations, particularly given elevated difficulties identifying and describing emotions observed in autism (Kinnaird et al. 2019). Nevertheless, we selected GAD‐7 for this study based on a combination of feedback from the autistic community (who ranked it equally to other, more autism‐specific tools, for example, Anxiety Scale for Autism‐Adults (ASA‐A) (Rodgers et al. 2020), also with a preference for its short format administration) and clinicians who utilize GAD‐7 routinely in practice. A high proportion (85%) of participants in this study further endorsed GAD‐7 as a relevant reflection of their anxiety experiences. Given the diverse experiences and preferences expressed by autistic people involved in this work regarding anxiety measurement, a combination of measures that includes autism‐specific/adapted tools (i.e., ASA‐A) may be optimal in future research and provide nuance in understanding the impact of intervention on dimensions of anxiety more specific to autism.

We also note that our sample was not representative of the autistic adult population. For instance, over two‐thirds of our participants were female, inverting the typically reported sex ratio for autism diagnosis and autism research samples [~2–5:1 male: female; (Lai et al. 2015)]. While an advantage in terms of capturing the experiences of autistic women, it is unclear whether our sample composition resulted from selection bias (i.e., autistic females perceiving this kind of research as more relevant/useful for them than males), increased barriers to participation for autistic males [e.g., stigma around mental health/help‐seeking; (Kauer et al. 2014)], increased barriers to access for traditional mental health support for autistic females (e.g., due to underdiagnosis and masking) that increase need for alternative support options, or other factors. Very few participants were from minority ethnic backgrounds and most were living independently, and in some form of part/full time education and/or employment. This reflects wider issues with the representativeness of research [and autism research, particularly research that is conducted remotely; please see (Rødgaard et al. 2022)] with diverse barriers to access reported in prior literature, including lack of trust in research (low perceived benefits and high perceived risks), burden (e.g., time, cost, and inaccessible format), misalignment between personal and research priorities, concerns about community perceptions of participation (stigma), and feeling unrepresented by lack of diversity in the research team (Woodall et al. 2010).

Additionally, the clinical content embedded in the tips and tools is delivered as written text and therefore relatively strong language and literacy skills are required for engagement. Some participants did find the need to remember to use the app, and/or aspects of engaging in the app (e.g., processing chunks of text information from tips and tools, entering and reliving worries) stressful. Accessibility can be improved through app optimization—for instance, providing more user control over the presentation of content (e.g., in terms of text size, color, and visual/audio prompts). We also identified (through both this study and wider feedback from public users) that some individuals and families have found the app most useful when used as a tool to promote reflection and discussions around anxiety with a support person present (i.e., a young person filling in the app with a parent; the app being used as a journal to form the basis for discussion with a clinical professional in therapy). This indicates potential additional areas for further research, in terms of the added value of digital intervention approaches like Molehill Mountain when embedded into existing/traditional therapeutic pathways. Indeed, participants commented on the importance that digital tools should not be viewed as a replacement for therapy, but rather a complementary approach that can make accessing therapy easier (and vice versa).

### 4.6 Conclusion

Here, we demonstrated that a novel app‐based anxiety management tool (“Molehill Mountain”) for autistic adults is feasible to implement (based on recruitment, retention, and intervention adherence), and acceptable to the community (based on user engagement and feedback). Furthermore, anxiety symptom severity was significantly reduced following the use of the app, on average, and this anxiety reduction was maintained for up to 6 months. We identified key areas (e.g., more frequent/tailored reminders to promote app engagement and greater personalization of features) for app optimization that would further increase the suitability of this tool to be tested under randomized controlled conditions in a full‐scale clinical trial, which participants of this study endorsed support for.

## Author Contributions

Conceptualization (study B.O. and E.S.; clinical components R.K. and A.O.), Investigation/Data curation (C.A.B. and B.O.), Formal analysis (B.O., C.A.B.; with support from D.R.), Funding acquisition (B.O. and E.S.), Methodology (B.O., E.S., C.A.B., B.C., S.B., A.H., and C.L.), Project administration (B.O., C.A.B., and E.S.), Resources (B.O. and A.R.), Visualization (B.O. and C.A.B.), writing – original draft (BO), writing – review and editing (all authors).

## Funding

This study received funding from the MRC Confidence in Concept award 2019 (1118148)‐awarded to B.O., E.S. E.S. and B.O. report grants during the conduct of (but unrelated to) this study from the Innovative Medicines Initiative 2 Joint Undertaking under grant agreement No 777394 for the project AIMS‐2‐TRIALS. This Joint Undertaking receives support from the European Union's Horizon 2020 research and innovation programme and EFPIA and SFARI, Autistica, Autism Speaks. The views expressed are those of the author(s) and not necessarily those of the NHS, MRC, nor IHI‐JU2. The funders had no role in the conceptualization of this study, nor the development of this publication.

## Conflicts of Interest

The authors declare no conflicts of interest.

## Supporting information


**Table S1:** Inclusion and exclusion criteria for study eligibility.
**Table S2:** App use experience survey responses at endpoint (T2).
**Table S3:** Baseline characteristics (T1) of those who completed endpoint (T2) vs. those who did not return (due to loss to follow up or withdrawal).
**Table S4:** Average results from Goal Attainment Scale ratings at baseline (T1) (goals selected, importance, distance from achieving goal), endpoint (T2), and follow ups 1, 2, and 3 (T3‐T5) (distance from achieving goal).

## Data Availability

Following funder and ethical guidelines, fully anonymised research data from this study will be made available via clinicaltrials.gov (Study Details|Molehill Mountain Feasibility Study|ClinicalTrials.gov) from participants who provided written consent to this in their consent forms.

## References

[aur70153-bib-0001] Adams, D. , and L.‐M. Emerson . 2021. “The Impact of Anxiety in Children on the Autism Spectrum.” Journal of Autism and Developmental Disorders 51, no. 6: 1909–1920. 10.1007/s10803-020-04673-3. 32876826

[aur70153-bib-0002] Ambrose, K. , D. Adams , K. Simpson , and D. Keen . 2020. “Exploring Profiles of Anxiety Symptoms in Male and Female Children on the Autism Spectrum.” Research in Autism Spectrum Disorders 76: 101601. 10.1016/j.rasd.2020.101601.

[aur70153-bib-0003] American Psychiatric Association, DSM‐5 Task Force . 2013. Diagnostic and Statistical Manual of Mental Disorders: DSM‐5™ (5th ed.). American Psychiatric Publishing, Inc. 10.1176/appi.books.9780890425596.

[aur70153-bib-0004] Association., A. P . 2013. Diagnostic and Statistical Manual of Mental Disorders (DSM‐V). 5th ed. American Psychiatric Association Publishing.

[aur70153-bib-0005] Backman, A. , L. Roll‐Pettersson , A. Mellblom , et al. 2024. “Internet‐Delivered Psychoeducation (SCOPE) for Transition‐Aged Autistic Youth: Pragmatic Randomized Controlled Trial.” Journal of Medical Internet Research 26: e49305. 10.2196/49305. 39608000 PMC11638691

[aur70153-bib-0006] Baumel, A. , K. Faber , N. Mathur , J. M. Kane , and F. Muench . 2017. “Enlight: A Comprehensive Quality and Therapeutic Potential Evaluation Tool for Mobile and Web‐Based eHealth Interventions.” Journal of Medical Internet Research 19, no. 3: e82. 10.2196/jmir.7270. 28325712 PMC5380814

[aur70153-bib-0007] Benevides, T. W. , S. M. Shore , M.‐L. Andresen , et al. 2020. “Interventions to Address Health Outcomes Among Autistic Adults: A Systematic Review.” Autism 24, no. 6: 1345–1359. 10.1177/1362361320913664. 32390461 PMC7787674

[aur70153-bib-0008] Bevan Jones, R. , F. Hussain , S. S. Agha , et al. 2023. “Digital Technologies to Support Adolescents With Depression and Anxiety: Review.” BJPsych Advances 29, no. 4: 239–253. 10.1192/bja.2022.3. 37521105 PMC10374830

[aur70153-bib-0009] Bird, G. , and R. Cook . 2013. “Mixed Emotions: The Contribution of Alexithymia to the Emotional Symptoms of Autism.” Translational Psychiatry 3: e285. 10.1038/tp.2013.61. 23880881 PMC3731793

[aur70153-bib-0010] Brede, J. , E. Cage , J. Trott , et al. 2022. “‘We Have to Try to Find a Way, a Clinical Bridge’—Autistic Adults' Experience of Accessing and Receiving Support for Mental Health Difficulties: A Systematic Review and Thematic Meta‐Synthesis.” Clinical Psychology Review 93: 102131. 10.1016/j.cpr.2022.102131. 35180632

[aur70153-bib-0011] Cadman, T. , H. Eklund , D. Howley , et al. 2012. “Caregiver Burden as People With Autism Spectrum Disorder and Attention‐Deficit/Hyperactivity Disorder Transition Into Adolescence and Adulthood in the United Kingdom.” Journal of the American Academy of Child & Adolescent Psychiatry 51, no. 9: 879–888. 10.1016/j.jaac.2012.06.017. 22917201

[aur70153-bib-0012] Catania, J. , S. Beaver , R. S. Kamath , et al. 2024. “Evaluation of Digital Mental Health Technologies in the United States: Systematic Literature Review and Framework Synthesis.” JMIR Ment Health 11: e57401. 10.2196/57401. 39213023 PMC11399741

[aur70153-bib-0013] Cooper, K. , M. E. Loades , and A. Russell . 2018. “Adapting Psychological Therapies for Autism.” Research in Autism Spectrum Disorders 45: 43–50. 10.1016/j.rasd.2017.11.002. 30245739 PMC6150418

[aur70153-bib-0014] Crane, L. , F. Adams , G. Harper , J. Welch , and E. Pellicano . 2018. “‘Something Needs to Change’: Mental Health Experiences of Young Autistic Adults in England.” Autism 23, no. 2: 477–493. 10.1177/1362361318757048. 29415558

[aur70153-bib-0015] Crane, L. , L. Goddard , and L. Pring . 2009. “Sensory Processing in Adults With Autism Spectrum Disorders.” Autism 13, no. 3: 215–228. 10.1177/1362361309103794. 19369385

[aur70153-bib-0016] den Houting, J. , D. Adams , J. Roberts , and D. Keen . 2019. “An Exploration of Autism‐Specific and Non‐Autism‐Specific Measures of Anxiety Symptomatology in School‐Aged Autistic Children.” Clinical Psychologist 23, no. 3: 237–248. 10.1111/cp.12174.

[aur70153-bib-0017] Doherty, M. , S. Neilson , J. Sullivan , et al. 2022. “Barriers to Healthcare and Self‐Reported Adverse Outcomes for Autistic Adults: A Cross‐Sectional Study.” BMJ Open 12, no. 2: e056904. 10.1136/bmjopen-2021-056904. PMC8883251 35193921

[aur70153-bib-0018] English, M. , G. Gignac , T. Visser , A. Whitehouse , J. Enns , and M. Maybery . 2021. “The Comprehensive Autistic Trait Inventory (CATI): Development and Validation of a New Measure of Autistic Traits in the General Population.” Molecular Autism 12, no. 1: 37. 10.1186/s13229-021-00475-1. 34001225 PMC8130295

[aur70153-bib-0019] Evans, D. W. , M. Uljarević , L. G. Lusk , E. Loth , and T. Frazier . 2017. “Development of Two Dimensional Measures of Restricted and Repetitive Behavior in Parents and Children.” Journal of the American Academy of Child & Adolescent Psychiatry 56, no. 1: 51–58. 10.1016/j.jaac.2016.10.014. 27993229

[aur70153-bib-0020] Gaigg, S. B. , P. E. Flaxman , G. McLaven , et al. 2020. “Self‐Guided Mindfulness and Cognitive Behavioural Practices Reduce Anxiety in Autistic Adults: A Pilot 8‐Month Waitlist‐Controlled Trial of Widely Available Online Tools.” Autism 24, no. 4: 867–883. 10.1177/1362361320909184. 32267168 PMC7418273

[aur70153-bib-0021] Gotham, K. , S. L. Bishop , S. Brunwasser , and C. Lord . 2014. “Rumination and Perceived Impairment Associated With Depressive Symptoms in a Verbal Adolescent–Adult ASD Sample.” Autism Research 7, no. 3: 381–391. 10.1002/aur.1377. 24802136 PMC4429601

[aur70153-bib-0022] Grist, R. , A. Croker , M. Denne , and P. Stallard . 2019. “Technology Delivered Interventions for Depression and Anxiety in Children and Adolescents: A Systematic Review and Meta‐Analysis.” Clinical Child and Family Psychology Review 22, no. 2: 147–171. 10.1007/s10567-018-0271-8. 30229343 PMC6479049

[aur70153-bib-0023] Gross, J. J. 2015. “Emotion Regulation: Current Status and Future Prospects.” Psychological Inquiry 26, no. 1: 1–26. 10.1080/1047840X.2014.940781.

[aur70153-bib-0024] Hollis, C. , C. J. Falconer , J. L. Martin , et al. 2017. “Annual Research Review: Digital Health Interventions for Children and Young People With Mental Health Problems: A Systematic and Meta‐Review.” Journal of Child Psychology and Psychiatry 58, no. 4: 474–503. 10.1111/jcpp.12663. 27943285

[aur70153-bib-0025] Hollocks, M. J. , J. W. Lerh , I. Magiati , R. Meiser‐Stedman , and T. S. Brugha . 2019. “Anxiety and Depression in Adults With Autism Spectrum Disorder: A Systematic Review and Meta‐Analysis.” Psychological Medicine 49, no. 4: 559–572. 10.1017/S0033291718002283. 30178724

[aur70153-bib-0026] Josyfon, E. , D. Spain , C. Blackmore , D. Murphy , and B. Oakley . 2023. “Alexithymia in Adult Autism Clinic Service‐Users: Relationships With Sensory Processing Differences and Mental Health.” Healthcare (Basel, Switzerland) 11, no. 24: 3114. 10.3390/healthcare11243114. 38132004 PMC10742835

[aur70153-bib-0027] Kauer, S. D. , C. Mangan , and L. Sanci . 2014. “Do Online Mental Health Services Improve Help‐Seeking for Young People? A Systematic Review.” Journal of Medical Internet Research 16, no. 3: e66. 10.2196/jmir.3103. 24594922 PMC3961801

[aur70153-bib-0028] Kerns, C. M. , P. C. Kendall , L. Berry , et al. 2014. “Traditional and Atypical Presentations of Anxiety in Youth With Autism Spectrum Disorder.” Journal of Autism and Developmental Disorders 44, no. 11: 2851–2861. 10.1007/s10803-014-2141-7. 24902932 PMC5441227

[aur70153-bib-0029] Kinnaird, E. , C. Stewart , and K. Tchanturia . 2019. “Investigating Alexithymia in Autism: A Systematic Review and Meta‐Analysis.” European Psychiatry 55: 80–89. 10.1016/j.eurpsy.2018.09.004. 30399531 PMC6331035

[aur70153-bib-0030] Kreslins, A. , A. E. Robertson , and C. Melville . 2015. “The Effectiveness of Psychosocial Interventions for Anxiety in Children and Adolescents With Autism Spectrum Disorder: A Systematic Review and Meta‐Analysis.” Child and Adolescent Psychiatry and Mental Health 9: 22. 10.1186/s13034-015-0054-7. 26120361 PMC4482189

[aur70153-bib-0032] Lai, M. C. , M. V. Lombardo , B. Auyeung , B. Chakrabarti , and S. Baron‐Cohen . 2015. “Sex/Gender Differences and Autism: Setting the Scene for Future Research.” Journal of the American Academy of Child and Adolescent Psychiatry 54, no. 1: 11–24. 10.1016/j.jaac.2014.10.003. 25524786 PMC4284309

[aur70153-bib-0031] Lai, M.‐C. , C. Kassee , R. Besney , et al. 2019. “Prevalence of Co‐Occurring Mental Health Diagnoses in the Autism Population: A Systematic Review and Meta‐Analysis.” Lancet. Psychiatry 6, no. 10: 819–829. 10.1016/S2215-0366(19)30289-5. 31447415

[aur70153-bib-0033] Lehtimaki, S. , J. Martic , Z. Rakonjac , N. Korać , O. Šerbić , and M. Blagojević . 2021. “Evidence on Digital Mental Health Interventions for Adolescents and Young People: Systematic Overview.” JMIR Mental Health 8, no. 4: e25847. 10.2196/25847. 33913817 PMC8120421

[aur70153-bib-0034] Li, C. E. , K. L. Wang , I. N. Treves , L. Bungert , J. D. E. Gabrieli , and L. Rozenkrantz . 2025. “Smartphone Mindfulness Intervention Reduces Anxiety Symptoms and Perceived Stress in Autistic Adults: A Randomized Controlled Trial.” Mindfulness 16, no. 6: 1504–1521. 10.1007/s12671-025-02558-z.

[aur70153-bib-0035] Lorenzo‐Luaces, L. , R. E. German , and R. J. DeRubeis . 2015. “It's Complicated: The Relation Between Cognitive Change Procedures, Cognitive Change, and Symptom Change in Cognitive Therapy for Depression.” Clinical Psychology Review 41: 3–15. 10.1016/j.cpr.2014.12.003. 25595660 PMC10080251

[aur70153-bib-0036] Maisel, M. E. , K. G. Stephenson , M. South , J. Rodgers , M. H. Freeston , and S. B. Gaigg . 2016. “Modeling the Cognitive Mechanisms Linking Autism Symptoms and Anxiety in Adults.” Journal of Abnormal Psychology 125, no. 5: 692–703. 10.1037/abn0000168. 27196436

[aur70153-bib-0037] Mason, D. , B. Ingham , A. Urbanowicz , et al. 2019. “A Systematic Review of What Barriers and Facilitators Prevent and Enable Physical Healthcare Services Access for Autistic Adults.” Journal of Autism and Developmental Disorders 49, no. 8: 3387–3400. 10.1007/s10803-019-04049-2. 31124030 PMC6647496

[aur70153-bib-0038] Mayes, S. D. , S. L. Calhoun , R. Aggarwal , et al. 2013. “Unusual Fears in Children With Autism.” Research in Autism Spectrum Disorders 7, no. 1: 151–158. 10.1016/j.rasd.2012.08.002.

[aur70153-bib-0039] Montazeri, F. , A. de Bildt , V. Dekker , and G. M. Anderson . 2019. “Network Analysis of Anxiety in the Autism Realm.” Journal of Autism and Developmental Disorders 49, no. 6: 2219–2230. 10.1007/s10803-018-3474-4. 29383649

[aur70153-bib-0040] National Autistic Society . 2019. “The Autism Act, 10 Years On: A Report from the All Party Parliamentary Group on Autism on Understanding, Services and Support for Autistic People and Their Families in England.”

[aur70153-bib-0041] Nicolaidis, C. , D. M. Raymaker , E. Ashkenazy , et al. 2015. “‘Respect the Way I Need to Communicate With You’: Healthcare Experiences of Adults on the Autism Spectrum.” Autism: The International Journal of Research and Practice 19, no. 7: 824–831. 10.1177/1362361315576221. 25882392 PMC4841263

[aur70153-bib-0042] Noel, K. , and B. Ellison . 2020. “Inclusive Innovation in Telehealth.” npj Digital Medicine 3: 89. 10.1038/s41746-020-0296-5. 32613084 PMC7316740

[aur70153-bib-0043] Oakley, B. , C. Boatman , S. Doswell , et al. 2023. “Molehill Mountain Feasibility Study: Protocol for a Non‐Randomised Pilot Trial of a Novel App‐Based Anxiety Intervention for Autistic People.” PLoS One 18, no. 7: e0286792. 10.1371/journal.pone.0286792. 37406026 PMC10321642

[aur70153-bib-0045] Oakley, B. , J. Tillmann , A. N. V. Ruigrok , et al. 2021. “COVID‐19 Health and Social Care Access for Autistic People: A European Policy Review.” BMJ Open 11: e045341. 10.1136/bmjopen-2020-045341. PMC8130751 34001500

[aur70153-bib-0046] Oakley, B. F. M. , E. J. H. Jones , D. Crawley , et al. 2020. “Alexithymia in Autism: Cross‐Sectional and Longitudinal Associations With Social‐Communication Difficulties, Anxiety and Depression Symptoms.” Psychological Medicine 52: 1–1470. 10.1017/S0033291720003244. PMC9226426 33028432

[aur70153-bib-0047] Patterson, B. , M. H. Boyle , M. Kivlenieks , and M. van Ameringen . 2016. “The Use of Waitlists as Control Conditions in Anxiety Disorders Research.” Journal of Psychiatric Research 83: 112–120. 10.1016/j.jpsychires.2016.08.015. 27585425

[aur70153-bib-0048] Rai, D. , D. Webb , A. Lewis , et al. 2024. “Sertraline for Anxiety in Adults With a Diagnosis of Autism (STRATA): Study Protocol for a Pragmatic, Multicentre, Double‐Blind, Placebo‐Controlled Randomised Controlled Trial.” Trials 25, no. 1: 37. 10.1186/s13063-023-07847-3. 38212784 PMC10782796

[aur70153-bib-0049] Ribaut, J. , A. DeVito Dabbs , F. Dobbels , et al. 2024. “Developing a Comprehensive List of Criteria to Evaluate the Characteristics and Quality of eHealth Smartphone Apps: Systematic Review.” JMIR mHealth and uHealth 12: e48625. 10.2196/48625. 38224477 PMC10825776

[aur70153-bib-0050] Riedelbauch, S. , S. B. Gaigg , T. Thiel , V. Roessner , and M. Ring . 2023. “Examining a Model of Anxiety in Autistic Adults.” Autism 28, no. 3: 565–579. 10.1177/13623613231177777. 37329157 PMC10913331

[aur70153-bib-0051] Robertson, A. E. , A. C. Stanfield , J. Watt , et al. 2018. “The Experience and Impact of Anxiety in Autistic Adults: A Thematic Analysis.” Research in Autism Spectrum Disorders 46: 8–18. 10.1016/j.rasd.2017.11.006.

[aur70153-bib-0052] Robeson, M. , K. M. Brasil , H. C. Adams , and K. R. Zlomke . 2024. “Measuring Depression and Anxiety in Autistic College Students: A Psychometric Evaluation of the PHQ‐9 and GAD‐7.” Autism 28, no. 11: 2793–2805. 10.1177/13623613241240183. 38514920

[aur70153-bib-0053] Rødgaard, E.‐M. , K. Jensen , K. W. Miskowiak , and L. Mottron . 2022. “Representativeness of Autistic Samples in Studies Recruiting Through Social Media.” Autism Research 15, no. 8: 1447–1456. 10.1002/aur.2777. 35809003 PMC9541916

[aur70153-bib-0054] Rodgers, J. , K. Farquhar , D. Mason , et al. 2020. “Development and Initial Evaluation of the Anxiety Scale for Autism‐Adults.” Autism in Adulthood 2, no. 1: 24–33. 10.1089/aut.2019.0044. 36600985 PMC8992845

[aur70153-bib-0055] Russell, A. , K. Cooper , S. Barton , et al. 2017. “Protocol for a Feasibility Study and Randomised Pilot Trial of a Low‐Intensity Psychological Intervention for Depression in Adults With Autism: The Autism Depression Trial (ADEPT).” BMJ Open 7, no. 12: e019545. 10.1136/bmjopen-2017-019545. PMC5736092 29203509

[aur70153-bib-0056] Shaw, S. C. K. , L. Carravallah , M. Johnson , et al. 2023. “Barriers to Healthcare and a ‘Triple Empathy Problem’ May Lead to Adverse Outcomes for Autistic Adults: A Qualitative Study.” Autism 28, no. 7: 1746–1757. 10.1177/13623613231205629. 37846479 PMC11191657

[aur70153-bib-0057] Sizoo, B. B. , and E. Kuiper . 2017. “Cognitive Behavioural Therapy and Mindfulness Based Stress Reduction May Be Equally Effective in Reducing Anxiety and Depression in Adults With Autism Spectrum Disorders.” Research in Developmental Disabilities 64: 47–55. 10.1016/j.ridd.2017.03.004. 28342404

[aur70153-bib-0058] South, M. , and J. Rodgers . 2017. “Sensory, Emotional and Cognitive Contributions to Anxiety in Autism Spectrum Disorders.” Frontiers in Human Neuroscience 11: 1–7. 10.3389/fnhum.2017.00020. 28174531 PMC5258728

[aur70153-bib-0059] Spaargaren, K. L. , Y. Roke , S. M. Begeer , et al. 2025. “A Randomized Controlled Trial Into the Effectiveness of a Mobile Health Application (SAM) to Reduce Stress and Improve Well‐Being in Autistic Adults.” Autism 29: 2588–2603. 10.1177/13623613251346885. 40567128 PMC12417602

[aur70153-bib-0060] Spain, D. , and F. Happé . 2020. “How to Optimise Cognitive Behaviour Therapy (CBT) for People With Autism Spectrum Disorders (ASD): A Delphi Study.” Journal of Rational‐Emotive & Cognitive‐Behavior Therapy 38, no. 2: 184–208. 10.1007/s10942-019-00335-1.

[aur70153-bib-0061] Spain, D. , J. Sin , T. Chalder , D. Murphy , and F. Happé . 2015. “Cognitive Behaviour Therapy for Adults With Autism Spectrum Disorders and Psychiatric Co‐Morbidity: A Review.” Research in Autism Spectrum Disorders 9: 151–162. 10.1016/j.rasd.2014.10.019.

[aur70153-bib-0062] Spek, A. A. , N. C. van Ham , and I. Nyklicek . 2013. “Mindfulness‐Based Therapy in Adults With an Autism Spectrum Disorder: A Randomized Controlled Trial.” Research in Developmental Disabilities 34, no. 1: 246–253. 10.1016/j.ridd.2012.08.009. 22964266

[aur70153-bib-0063] Spitzer, R. L. , K. Kroenke , J. B. W. Williams , and B. Löwe . 2006. “A Brief Measure for Assessing Generalized Anxiety Disorder: The GAD‐7.” Archives of Internal Medicine 166, no. 10: 1092–1097. 10.1001/archinte.166.10.1092. 16717171

[aur70153-bib-0064] Sukhodolsky, D. G. , M. H. Bloch , K. E. Panza , and B. Reichow . 2013. “Cognitive‐Behavioral Therapy for Anxiety in Children With High‐Functioning Autism: A Meta‐Analysis.” Pediatrics 132, no. 5: e1341–e1350. 10.1542/peds.2013-1193. 24167175 PMC3813396

[aur70153-bib-0065] Sutherland, R. , D. Trembath , and J. Roberts . 2018. “Telehealth and Autism: A Systematic Search and Review of the Literature.” International Journal of Speech‐Language Pathology 20, no. 3: 324–336. 10.1080/17549507.2018.1465123. 29709201

[aur70153-bib-0066] Tromans, S. , W. Henley , I. Summers , et al. 2023. “The Psychological and Social Impact of the Digital Self‐Support System ‘Brain in Hand’ on Autistic People: Prospective Cohort Study in England and Wales.” BJPsych Open 9, no. 3: e96. 10.1192/bjo.2023.57. 37232106 PMC10228225

[aur70153-bib-0067] Ung, D. , R. Selles , B. J. Small , and E. A. Storch . 2015. “A Systematic Review and Meta‐Analysis of Cognitive‐Behavioral Therapy for Anxiety in Youth With High‐Functioning Autism Spectrum Disorders.” Child Psychiatry & Human Development 46, no. 4: 533–547. 10.1007/s10578-014-0494-y. 25246292

[aur70153-bib-0068] van Steensel, F. J. A. , S. M. Bögels , and C. D. Dirksen . 2012. “Anxiety and Quality of Life: Clinically Anxious Children With and Without Autism Spectrum Disorders Compared.” Journal of Clinical Child & Adolescent Psychology 41, no. 6: 731–738. 10.1080/15374416.2012.698725. 22775580

[aur70153-bib-0069] Walters, S. , M. Loades , and A. Russell . 2016. “A Systematic Review of Effective Modifications to Cognitive Behavioural Therapy for Young People With Autism Spectrum Disorders.” Review Journal of Autism and Developmental Disorders 3, no. 2: 137–153. 10.1007/s40489-016-0072-2.

[aur70153-bib-0070] Wood, J. J. , P. C. Kendall , K. S. Wood , et al. 2020. “Cognitive Behavioral Treatments for Anxiety in Children With Autism Spectrum Disorder: A Randomized Clinical Trial.” JAMA Psychiatry 77, no. 5: 474–483. 10.1001/jamapsychiatry.2019.4160. 31755906 PMC6902190

[aur70153-bib-0071] Woodall, A. , C. Morgan , C. Sloan , and L. Howard . 2010. “Barriers to Participation in Mental Health Research: Are There Specific Gender, Ethnicity and Age Related Barriers?” BMC Psychiatry 10, no. 1: 103. 10.1186/1471-244X-10-103. 21126334 PMC3016310

[aur70153-bib-0072] World Health Organisation . 2023. “Anxiety Disorders.” September 27. https://www.who.int/news‐room/fact‐sheets/detail/anxiety‐disorders.

[aur70153-bib-0073] Yang, Y. J. , and K.‐M. Chung . 2022. “Pilot Randomized Control Trial of an App‐Based CBT Program for Reducing Anxiety in Individuals With ASD Without Intellectual Disability.” Journal of Autism and Developmental Disorders 53: 1331–1346. 10.1007/s10803-022-05617-9. 35689137

[aur70153-bib-0074] Zigmond, A. S. , and R. P. Snaith . 1983. “The Hospital Anxiety and Depression Scale.” Acta Psychiatrica Scandinavica 67, no. 6: 361–370. 10.1111/j.1600-0447.1983.tb09716.x. 6880820

