# Supplementary material for: Feasibility Study of a Novel App‐Based Anxiety Intervention for Autistic People
Source: Autism Res. 2025 Dec 14;19(1):e70153. doi: 10.1002/aur.70153 (PMC12853239; doi:10.1002/aur.70153)
Supplement: Supplementary file 1 — Table S1: Inclusion and exclusion criteria for study eligibility. Table S2: App use experience survey responses at endpoint (T2). Table S3: Baseline characteristics (T1) of those who completed endpoint (T2) vs. those who did not return (due to loss to follow up or withdrawal). Table S4: Average results from Goal Attainment Scale ratings at baseline (T1) (goals selected, importance, distance from achieving goal), endpoint (T2), and follow ups 1, 2, and 3 (T3‐T5) (distance from achieving goal). [file AUR-19-0-s001.docx]

Supplementary Table 1. *Inclusion and exclusion criteria for study eligibility.*

| **Inclusion criteria** |
| --- |
| Age ≥ 16-years. |
| Autism diagnosis. |
| Current mild-to-severe anxiety symptom severity, as assessed at screening. |
| Able and willing to provide verbal and written informed consent to take part in the study. Fluency in verbal/ written English for the purposes of engaging with the app materials. |
| Able and willing to use a mobile phone app. |
| **Exclusion criteria** |
| Difficulties with reading/ writing to the extent that the app is inaccessible (i.e., able to use the mobile phone app). |
| High risk of self-harm that make participation in the study inappropriate for the individual’s current level of clinical need (as assessed by clinical team). |
| Attended ≥ 6 sessions of individual or group therapy (e.g., cognitive behavioural therapy) in the past 6-months, which would make it impossible to parse out the effects of the app from existing therapy. |
| If using psychotropic medication, this medication/ dose must have been stable for a minimum of 8-weeks on entry to the study. |

Supplementary Table 2. *App use experience survey responses at endpoint (T2).*

| Question | Total responses | Response options | Agreement | |
| --- | --- | --- | --- | --- |
|  | N |  | N | % |
| 1. During this study, how often did you use Molehill Mountain, on average? | 72 | Every day (1) | 12 | 17 |
|  |  | Most days (4-6 days per week) (2) | 31 | 43 |
|  |  | Some days (1-3 days per week) (3) | 9 | 13 |
|  |  | Infrequently (not every week) (4) | 11 | 15 |
|  |  | Rarely (only a few times) (5) | 8 | 11 |
|  |  | Never (6) | 1 | 1 |
|  |  | Not sure/Prefer not to answer (7) | 0 | 0 |
| 2. Did you completely stop using Molehill Mountain before the 13-weeks of the study were up? | 72 | Yes (1) | 23 | 32 |
|  |  | No (2) | 47 | 65 |
|  |  | Not sure/prefer not to say (3) | 2 | 3 |
| 2b. If Y to Q2: When did you stop using Molehill Mountain? | 23 | Within 1 week (1) | 3 | 13 |
|  |  | After 1-2 weeks (2) | 3 | 13 |
|  |  | After 3-4 weeks (3) | 5 | 22 |
|  |  | After 5-6 weeks (4) | 5 | 22 |
|  |  | After 7-8 weeks (5) | 3 | 13 |
|  |  | After 9-10 weeks (6) | 0 | 0 |
|  |  | After 11-12 weeks (7) | 3 | 13 |
|  |  | Not sure/ Prefer not to answer (8) | 1 | 3 |
| 2c. If Y to Q2: Why did you stop using Molehill Mountain?* | 23 | Didn't like the app (1) | 4 | 17 |
|  |  | Technical issues with the app (2) | 6 | 26 |
|  |  | Forgot to keep using the app (3) | 9 | 39 |
|  |  | Takes too long (4) | 1 | 3 |
|  |  | Personal reasons/ change in circumstance (5) | 3 | 13 |
|  |  | Didn't find the app was helping with my anxiety (6) | 10 | 43 |
|  |  | My anxiety improved, so I felt I didn't need the app anymore (7) | 1 | 3 |
|  |  | Other – Please Specify (8) | 6 | 26 |
|  |  | Not sure/ Prefer not to answer (9) | 0 | 0 |
| 3. During the study, how long did you use Molehill Mountain for at each check in, on average? | 71 | Less than 5 minutes (1) | 34 | 48 |
|  |  | 5-15 minutes (2) | 36 | 51 |
|  |  | 15-30 minutes (3) | 1 | 1 |
|  |  | 30-45 minutes (4) | 0 | 0 |
|  |  | 45-60 minutes (5) | 0 | 0 |
|  |  | More than 60 minutes (6) | 0 | 0 |
|  |  | Not sure/Prefer not to answer (7) | 0 | 0 |
| 4. If you used Molehill Mountain intermittently, what factors made you use it again after a period of less/ no use? | 41 | I had sometimes forgotten about using the app, but then remembered (1) | 24 | 59 |
|  |  | I had more time to use the app than previously (2) | 3 | 7 |
|  |  | I used the app as and when I needed to (e.g., at times of higher anxiety only) (3) | 1 | 2 |
|  |  | Change in personal circumstance meant I could use the app more than previously (4) | 0 | 0 |
|  |  | I wanted to give the app another chance after deciding not to continue with it (5) | 0 | 0 |
|  |  | Not applicable: I did not use the app intermittently (6) | 6 | 15 |
|  |  | Other - Please specify (7) | 6 | 15 |
|  |  | Not sure/ Prefer not to answer (8) | 1 | 2 |
| 5. What would help you to use Molehill Mountain more, or for longer, in future? Try to be as specific as you can, to help us improve the app.** | - | - | - | - |
| 6. How easy did you find Molehill Mountain to use overall? | 72 | Very easy (1) | 22 | 31 |
|  |  | Easy (2) | 30 | 42 |
|  |  | Neither easy, nor difficult (3) | 13 | 18 |
|  |  | Difficult (4) | 5 | 7 |
|  |  | Very difficult (5) | 0 | 0 |
|  |  | Not sure/ Prefer not to answer (6) | 2 | 3 |
| 6b. What would make the app easier to use?** | - | - | - | - |
| 7. Did you experience any technical issues with the app? | 72 | Yes (1) | 21 | 29 |
|  |  | No (2) | 49 | 68 |
|  |  | Not sure/ Prefer not to answer (3) | 2 | 3 |
| 7b. If Y to Q7: what technical issues did you experience with the app?** | - | - | - | - |
| 8. Please rank the following app features in order of how useful/ relevant they were to you from Most Useful, to Least Useful:  Daily check in function | 32 | Most useful (1) | 14 | 44 |
|  |  | (2) | 10 | 31 |
|  |  | (3) | 2 | 6 |
|  |  | (4) | 2 | 6 |
|  |  | (5) | 3 | 9 |
|  |  | Least useful (6) | 1 | 3 |
| 8. Worry Mountain | 32 | Most useful (1) | 6 | 19 |
|  |  | (2) | 3 | 9 |
|  |  | (3) | 5 | 16 |
|  |  | (4) | 8 | 25 |
|  |  | (5) | 5 | 16 |
|  |  | Least useful (6) | 5 | 16 |
| 8. Breathing/ relaxation exercises | 32 | Most useful (1) | 1 | 3 |
|  |  | (2) | 5 | 16 |
|  |  | (3) | 6 | 19 |
|  |  | (4) | 7 | 22 |
|  |  | (5) | 8 | 25 |
|  |  | Least useful (6) | 5 | 16 |
| 8. Anxiety tips and tools | 32 | Most useful (1) | 5 | 16 |
|  |  | (2) | 9 | 28 |
|  |  | (3) | 8 | 25 |
|  |  | (4) | 4 | 13 |
|  |  | (5) | 3 | 9 |
|  |  | Least useful (6) | 3 | 9 |
| 8. Being able to see your stats/progress in the app | 32 | Most useful (1) | 2 | 6 |
|  |  | (2) | 2 | 6 |
|  |  | (3) | 5 | 16 |
|  |  | (4) | 8 | 25 |
|  |  | (5) | 4 | 13 |
|  |  | Least useful (6) | 11 | 34 |
| 8. Diary function | 32 | Most useful (1) | 4 | 13 |
|  |  | (2) | 3 | 9 |
|  |  | (3) | 6 | 19 |
|  |  | (4) | 3 | 9 |
|  |  | (5) | 9 | 28 |
|  |  | Least useful (6) | 7 | 22 |
| 9. Are there any additional features that you would suggest for the app in the future? If yes, please detail them here** | - | - | - | - |
| 10. To what extent was the visual design of the app appealing (e.g., aesthetics, layout, size?) | 42 | Very appealing (1) | 9 | 21 |
|  |  | Quite appealing (2) | 19 | 45 |
|  |  | A little appealing (3) | 7 | 17 |
|  |  | Not very appealing (4) | 7 | 17 |
|  |  | Not sure/ Prefer not to answer (5) | 0 | 0 |
| 11. Do you have any data privacy or security concerns around using an anxiety app like Molehill Mountain? | 42 | No (1) | 34 | 81 |
|  |  | Yes (2) | 6 | 14 |
|  |  | Not sure/Prefer not to answer (3) | 2 | 5 |
| 12. Have you tried any other anxiety apps, except from Molehill Mountain? | 43 | Yes, before taking part in this study only (1) | 15 | 35 |
|  |  | Yes, before taking part in this study and since joining this study (2) | 2 | 5 |
|  |  | Yes, since joining this study only (3) | 0 | 0 |
|  |  | No (4) | 24 | 56 |
|  |  | Not sure/ Prefer not to answer (5) | 2 | 5 |
| 12b. If Y to Q12: Please specify which other anxiety apps you use/ have used (aside from Molehill Mountain).** | - | - | - | - |
| 12c. If Y to Q12: How would you compare your experiences of using the Molehill Mountain app with other anxiety apps you have used/ are using? | 5 | I prefer Molehill Mountain (1) | 0 | 0 |
|  |  | I prefer another/ other anxiety apps over Molehill Mountain - Please specify (2) | 1 | 20 |
|  |  | I have no preference between Molehill Mountain and another/ other anxiety apps (3) | 3 | 60 |
|  |  | Not sure/ Prefer not to answer (4) | 1 | 20 |
| 12d. If Y to Q12: What are the main reasons for your app preference?** | - | - | - | - |
| 13. Do you feel that an app is a good format for offering anxiety support to autistic people? | 41 | Yes, definitely - Please specify (1) | 14 | 34 |
|  |  | Yes, to some extent - Please specify (2) | 16 | 39 |
|  |  | No - Please specify (3) | 8 | 20 |
|  |  | Not sure/ Prefer not to answer (4) | 3 | 7 |
| 14. How much did Molehill Mountain help you to understand your anxiety? | 71 | Not at all (1) | 17 | 24 |
|  |  | A little (2) | 24 | 34 |
|  |  | Quite a lot (3) | 21 | 30 |
|  |  | Very much (4) | 7 | 10 |
|  |  | Not sure/ Prefer not to answer (5) | 2 | 3 |
| 15. How much did Molehill Mountain help you manage your anxiety? | 71 | Not at all (1) | 21 | 30 |
|  |  | A little (2) | 37 | 52 |
|  |  | Quite a lot (3) | 6 | 8 |
|  |  | Very much (4) | 3 | 4 |
|  |  | Not sure/ Prefer not to answer (5) | 4 | 6 |
| 16. In what ways do you feel Molehill Mountain helped you?* | 71 | I understand my anxiety more (1) | 22 | 31 |
|  |  | I understand my anxiety triggers more (2) | 25 | 35 |
|  |  | I can tell when I am getting anxious more easily (3) | 13 | 18 |
|  |  | I can describe my anxiety better to others (4) | 19 | 27 |
|  |  | I can manage my anxiety triggers better (5) | 10 | 14 |
|  |  | I can manage my anxiety better (6) | 7 | 10 |
|  |  | My anxiety is reduced (7) | 9 | 13 |
|  |  | Anxiety has less impact on my everyday life (8) | 9 | 13 |
|  |  | Anxiety has less impact on my overall wellbeing (9) | 6 | 8 |
|  |  | Other symptoms, like depression or irritability, have reduced (10) | 3 | 4 |
|  |  | My social life has improved (11) | 14 | 20 |
|  |  | There has been no benefit to me (12) | 8 | 11 |
|  |  | Other - Please specify (13) | 6 | 8 |
|  |  | Not sure/ Prefer not to answer (14) | 11 | 15 |
| 17. Which kinds of anxiety do you feel Molehill Mountain helped with?* | 41 | General anxiety (e.g. worrying about lots of different things, etc) (1) | 28 | 68 |
|  |  | Social anxiety (e.g. speaking in public, meeting new people, etc) (2) | 12 | 29 |
|  |  | Agoraphobic anxiety (e.g. leaving house, being in crowded or public spaces, etc) (3) | 1 | 2 |
|  |  | Specific fears/ phobias (e.g. fear of water, fear of spiders, etc) (4) | 8 | 20 |
|  |  | Panic attacks (5) | 4 | 10 |
|  |  | Post-traumatic stress (6) | 2 | 5 |
|  |  | Obsessive compulsive anxiety (e.g., unwanted thoughts/behaviours that happen over and over) (7) | 5 | 12 |
|  |  | Other – Please specify (8) | 1 | 2 |
|  |  | None of the above (9) | 8 | 20 |
|  |  | Not sure/ Prefer not to answer (10) | 3 | 7 |
| 18. Did using Molehill Mountain change your experiences of any of the following features that may be related to anxiety in some cases?* | 41 | Repetitive behaviours (e.g., stimming, soothing) decreased (1) | 4 | 10 |
|  |  | Repetitive behaviours (e.g., stimming, soothing) increased (2) | 1 | 2 |
|  |  | ‘Masking’ or camouflaging of autistic traits decreased (3) | 10 | 24 |
|  |  | ‘Masking’ or camouflaging of autistic traits increased (4) | 1 | 2 |
|  |  | Meltdowns/ shutdowns decreased (5) | 6 | 15 |
|  |  | Meltdowns/ shutdowns increased (6) | 1 | 2 |
|  |  | Sensory sensitivities decreased (7) | 1 | 2 |
|  |  | Sensory sensitivities increased (8) | 1 | 2 |
|  |  | Feelings of social isolation/ loneliness decreased (9) | 3 | 7 |
|  |  | Feelings of social isolation/ loneliness increased (10) | 3 | 7 |
|  |  | Getting ‘stuck’ in cycles of thoughts decreased (11) | 13 | 32 |
|  |  | Getting ‘stuck’ in cycles of thoughts increased (12) | 2 | 5 |
|  |  | I understand my own emotions better (13) | 18 | 44 |
|  |  | I understand the emotions of others better (14) | 4 | 10 |
|  |  | Other - Please specify (15) | 4 | 10 |
|  |  | None of the above (16) | 9 | 22 |
|  |  | Not sure/ Prefer not to answer (17) | 1 | 2 |
| 19. Were there any features of Molehill Mountain that definitely did not help with anxiety/ increased anxiety? | 42 | Yes (1) | 17 | 40 |
|  |  | No (2) | 18 | 43 |
|  |  | Prefer not to answer (3) | 7 | 17 |
| 19b. Which features did not help with anxiety, or increased anxiety?** | - | - | - | - |
| 20. Are there any items/skills in the app that you will now use/do now use in your daily life to help with your anxiety?** | - | - | - | - |
| 21. Having used the app, do you think any of the tips provided will help you prevent or manage your anxiety in the future? | 40 | Yes (1) | 27 | 68 |
|  |  | No (2) | 13 | 33 |
| 22. Would you keep using Molehill Mountain in the future? | 70 | Definitely (1) | 10 | 14 |
|  |  | Likely (2) | 22 | 31 |
|  |  | Maybe (3) | 18 | 26 |
|  |  | No, but I might consider other anxiety apps (4) | 8 | 11 |
|  |  | No (5) | 12 | 17 |
|  |  | Not sure/ Prefer not to answer (6) | 0 | 0 |
| 23. Were the anxiety questionnaires used in this study appropriate/ relevant to your experiences? | 42 | Very much (1) | 14 | 33 |
|  |  | Somewhat (2) | 22 | 52 |
|  |  | Not too much (3) | 4 | 10 |
|  |  | Not at all (4) | 1 | 2 |
|  |  | Not sure/ Prefer not to answer (5) | 1 | 2 |
| 24. Did you find any of the questions study questionnaires distressing to answer? | 41 | Yes (1) | 4 | 10 |
|  |  | No (2) | 37 | 90 |
| 24b. If Y to Q24: Which questions did you find particularly distressing?** | - | - | - | - |
| 24c. If Y to Q24: What was it about those questions that were distressing?** | - | - | - | - |
| 24d. If Y to Q24: How can we make this less distressing in the future?** | - | - | - | - |
| 25. How did you find the recommended 13-week time period for using Molehill Mountain in this study? | 42 | Too little time/ not long enough (1) | 2 | 5 |
|  |  | Too much time/ too long (2) | 15 | 36 |
|  |  | About the right amount of time (3) | 19 | 45 |
|  |  | Not sure/ Prefer not to answer (4) | 6 | 14 |
| 26. If there were a clinical trial of a mobile app to support autistic people with anxiety, would you ever consider taking part?*** | 70 | Yes (1) | 66 | 94 |
|  |  | No (2) | 1 | 1 |
|  |  | Not sure/ Prefer not to answer (3) | 3 | 4 |
| 27. If you were to take part in a clinical trial of a mobile app to support autistic people with anxiety, would you be happy to be randomised?**** | 71 | Yes (1) | 50 | 70 |
|  |  | Yes, but only if I could access the mobile app after the trial was finished (in case I am in the comparison group) (2) | 16 | 23 |
|  |  | No (3) | 2 | 3 |
|  |  | Not sure/ Prefer not to answer (4) | 3 | 4 |
| 28. How did using the app compare to any other strategies you use/ therapy you have received in the past (e.g online/in person therapy)?** | - | - | - | - |
| 29. Do you have any other suggestions about what you would most like to see in an app like Molehill Mountain in the future? What should we do next after this study?** | - | - | - | - |

Note: *Multiple choice option – more than one response possible per participant. **Questions that required free text responses are listed for reference but free text comments (and open-ended questions only resulting in free text responses) are not included for ease of interpretation. ***Additional/guidance notes on this question in the survey stated: “*A clinical trial is a research study that compares whether a specific intervention (i.e., a mobile app for anxiety) leads to more improvements in anxiety, compared with another type of intervention (e.g., face-to-face therapy), or no intervention at all. This is to see whether the mobile app is really effective at doing what it is supposed to do – reducing anxiety.”* ****Additional/guidance notes on this question in the survey stated: *“Randomisation is where one group of people in a clinical trial receive the intervention being tested (i.e., the mobile app). A second group of people in the trial receive either a different type of intervention, or no intervention at all. This is so that the intervention being tested can be directly compared to something, to make sure that it really is effective. It’s called randomisation, because which group you end up in is picked completely at random.”*

Supplementary Table 3. *Baseline characteristics (T1) of those who completed endpoint (T2) vs. those who did not return (due to loss to follow up or withdrawal).*

|  |  | **Completed endpoint (T2)** | | | **Lost to follow up** | | | **Comparison** | |
| --- | --- | --- | --- | --- | --- | --- | --- | --- | --- |
|  |  | ***N*** |  |  | ***N*** |  |  | **φ / φc** | **p** |
| **Demographics** | **Sex (Female:Male)*** | 54:22 |  |  | 15:8 |  |  | 0.05 | 0.59 |
|  | **Gender identity** |  |  |  |  |  |  |  |  |
|  | Female | 59 |  |  |  |  |  |  |  |
|  | Male | 31 |  |  |  |  |  |  |  |
|  | Third gender/non-binary | 2 |  |  |  |  |  |  |  |
|  | Other (self-describe)** | 2 |  |  |  |  |  |  |  |
|  | Prefer not to answer | 5 |  |  |  |  |  |  |  |
|  | **Ethnicity** |  |  |  |  |  |  |  |  |
|  | White | 68 |  |  | 20 |  |  | 0.14 | 0.62 |
|  | More than one | 3 |  |  | 1 |  |  |  |  |
|  | Black | 1 |  |  | 1 |  |  |  |  |
|  | Asian | 1 |  |  | 0 |  |  |  |  |
|  | Other (self-describe)** | 1 |  |  | 1 |  |  |  |  |
|  | Prefer not to answer | 2 |  |  | 0 |  |  |  |  |
|  | **Living arrangements** |  |  |  |  |  |  |  |  |
|  | Independently | 58 |  |  | 12 |  |  | 0.36 | 0.02 |
|  | Parents/family | 15 |  |  | 6 |  |  |  |  |
|  | Daily/regular support | 1 |  |  | 3 |  |  |  |  |
|  | Prefer not to answer | 2 |  |  | 2 |  |  |  |  |
|  | **Education/employment** |  |  |  |  |  |  |  |  |
|  | Full-time | 36 |  |  | 11 |  |  | 0.38 | 0.66 |
|  | Part-time | 18 |  |  | 3 |  |  |  |  |
|  | Voluntary | 6 |  |  | 1 |  |  |  |  |
|  | Not in | 13 |  |  | 6 |  |  |  |  |
|  | Retired or carer | 3 |  |  | 2 |  |  |  |  |
|  | **Co-occurring diagnoses** |  |  |  |  |  |  |  |  |
|  | Anxiety | 64 |  |  | 22 |  |  | 0.14 | 0.15 |
|  | Depression | 52 |  |  | 15 |  |  | -0.03 | 0.77 |
|  | Specific learning difficulties | 20 |  |  | 4 |  |  | -0.09 | 0.38 |
|  | ADHD | 14 |  |  | 7 |  |  | 0.12 | 0.22 |
|  | Eating disorders | 13 |  |  | 3 |  |  | 0.03 | 0.75 |
|  | OCD | 11 |  |  | 2 |  |  | -0.07 | 0.47 |
|  | Tics/Tourette’s | 2 |  |  | 2 |  |  | 0.13 | 0.20 |
|  | DCD | 2 |  |  | 0 |  |  | 0.08 | 0.43 |
|  | Psychosis | 2 |  |  | 0 |  |  | -0.08 | 0.43 |
|  | Bipolar | 1 |  |  | 1 |  |  | =0.09 | 0.37 |
|  | Conduct problems | 1 |  |  | 0 |  |  | -0.06 | 0.58 |
|  | **Medications** | 46 |  |  | 13 |  |  | 0.02 | 0.82 |
|  | **Therapy (past 6 months)** | 8 |  |  | 4 |  |  | -0.16 | 0.11 |
|  |  | ***N*** | **Mean (SD)** | **Range** | ***N*** | **Mean (SD)** | **Range** | ***d [95% CI]*** | ***p*** |
|  | **Age (years)** | 76 | 41.64 (13.54) | 16-74 | 23 | 38.61 (15.62) | 16-65 | -0.22 [-0.68, 0.25] | 0.41 |
|  | **Age of autism dx** | 76 | 34.86 (15.99) | 4-69 | 23 | 32.52 (15.76) | 8-56 | -0.15 [-0.61,0.32] | 0.54 |
| **Autistic traits** | **CAT-I (Total)** | 70 | 156.39 (21.63) | 79-183 | 19 | 161.47 (18.73) | 121-193 | 0.24 [-0.27,0.75] | 0.32 |
|  | **CAT-I (Social)** | 70 | 26.74 (5.06) | 13-35 | 19 | 26.11 (3.20) | 19-31 | -0.13 [-0.64, 0.38] | 0.51 |
|  | **CAT-I (Communication)** | 70 | 24.11 (4.62) | 10-33 | 19 | 24.16 (4.66) | 16-31 | 0.01 [-0.50, 0.52] | 0.97 |
|  | **CAT-I (Camouflage)** | 70 | 24.90 (4.69) | 11-33 | 19 | 26.32 (2.94) | 22-32 | 0.32 [-0.19, 0.83] | 0.11 |
|  | **CAT-I (Rigidity)** | 70 | 27.66 (5.32) | 11-35 | 19 | 29.63 (3.48) | 23-35 | 0.39 [-0.12, 0.90] | 0.06 |
|  | **CAT-I (RRB)** | 70 | 25.24 (5.31) | 11-34 | 19 | 27.79 (3.08) | 20-33 | 0.52 [0.00, 1.03] | 0.01 |
|  | **CAT-I (Sensory)** | 70 | 27.73 (6.30) | 8-35 | 19 | 27.47 (6.00) | 16-35 | -0.04 [-0.55, 0.47] | 0.87 |
|  | **ARI-R (Total)** | 70 | 186.01 (35.05) | 80-247 | 19 | 204.26 (27.95) | 154-265 | 0.54 [0.03, 1.05] | 0.02 |
| **Anxiety (and depression)** | **GAD-7 (Total)***** | 76 | 13.84 | 5-21 | 24 | 11.29 (4.79) | 5-21 | -0.57 [-1.03, -0.10] | 0.03 |
|  | **HADS (Anxiety)** | 70 | 14.21 (3.53) | 6-21 | 19 | 16.47 (2.21) | 14-21 | 0.69 [0.17, 1.20] | <0.01 |
|  | **HADS (Depression)** | 70 | 9.59 (4.36) | 0-19 | 19 | 8.68 (5.00) | 0-19 | -0.20 [-0.71, 0.31] | 0.48 |

*Note*. *Represents sex at birth. **Ethnicity: Jewish (n=1), Irish (n=1). ***Core anxiety measure (assessed during screening to confirm inclusion criteria). *N*=Number of participants, φ=phi effect size, φc=Cramer’s v effect size, *p*=p-value for significance, *d* [95% CI]=Cohen’s d effect size with 95% confidence intervals, dx=diagnosis, ADHD=Attention deficit hyperactivity disorder; OCD=Obsessive-compulsive disorder; DCD=Developmental Coordination Disorder; CAT-I=Comprehensive Autistic Trait Inventory, CAT-I (RRB)= Comprehensive Autistic Trait Inventory Restricted and Repetitive Behaviours Subscale, ARI-R=Adult Routines Inventory – Revised, GAD-7=Generalised Anxiety Disorder – 7 item scale, HADS=Hospital Anxiety and Depression Scale.

Supplementary Table 4. *Average results from Goal Attainment Scale ratings at baseline (T1) (goals selected, importance, distance from achieving goal), endpoint (T2), and follow ups 1, 2, and 3 (T3-T5) (distance from achieving goal).*

| **Goal** | **N times selected (%*)** | **Average (range) importance rating**  1. Not at all important  2. A little important  3. Moderately important  4. Very important | **Average distance from goal**  1. A long way from this goal  2. A little way from this goal  3. I have achieved this goal  4. I have exceeded this goal  5. I have exceeded this goal by a lot | | | | |  |
| --- | --- | --- | --- | --- | --- | --- | --- | --- |
|  | **T1** | **T1** | **T1** | **T2** | **T3** | **T4** | **T5** | |
| 1. I would like to feel less anxious about communicating over the phone (e.g., with professionals, with friends, in front of others, etc) and/or answering the front door. | 18 (20%) | 3 (1-4) | 1 (1-2) | 2 (1-5)* | 2 (1-3)* | 2 (1-3)* | 2 (1-3)* | |
| 2. I would like to feel less anxious when myself or somebody else is not following the rules. | 12 (14%) | 3 (2-4) | 2 (1-2) | 2 (1-3) | 1 (1-2) | 2 (1-2) | 2 (1-3) | |
| 3. I would like to feel less anxious about change (e.g., learning new ways to do things, disruptions in routine/plan, when something is out of order or out of place, etc). | 36 (41%) | 3 (2-4) | 1 (1-3) | 2 (1-4)* | 1 (1-2) | 2 (1-3)* | 2 (1-3)* | |
| 4. I would like to feel less anxious about different sensory environments or triggers (e.g., lots of lights or music, screaming children, loud noises, multiple people talking, etc). | 23 (26%) | 3 (2-4) | 1 (1-2) | 2 (1-3)* | 2 (1-3)* | 2 (1-2)* | 2 (1-3)* | |
| 5. I would like to feel less anxious about social situations, being out in public, communicating, and talking to others. | 37 (42%) | 4 (2-4) | 1 (1-2) | 2 (1-3)* | 2 (1-3)* | 2 (1-2)* | 2 (1-3)* | |
| 6. I would like to feel less anxious when meeting new people or making new friends. | 11 (13%) | 3 (2-4) | 1 (1-2) | 2 (1-4)* | 1 (1-3) | 2 (1-2)* | 2 (1-2)* | |
| 7. I would like to feel more confident to mask less and be myself in social situations (e.g., speaking more about my interests). | 16 (18%) | 3 (2-4) | 2 (1-3) | 2 (1-2) | 2 (1-2) | 2 (1-3) | 2 (1-3) | |
| 8. I would like to be more assertive and to feel less anxious about speaking up in social situations, such as asking questions and giving an opinion. | 14 (16%) | 4 (3-4) | 1 (1-2) | 2 (1-3)* | 2 (1-3)* | 1 (1-2) | 1 (1-2) | |
| 9. I would like to feel less anxious in my relationships (e.g., with friends, family, partner). | 13 (15%) | 4 (2-4) | 1 (1-2) | 2 (1-2)* | 1 (1-2) | 2 (1-3)* | 2 (1-5)* | |
| 10. I would like to feel less anxious when approaching unknown or new situations. | 12 (14%) | 4 (3-4) | 1 (1-2) | 1 (1-2) | 1 (1-2) | 2 (1-3)* | 2 (1-3)* | |
| 11. I would like to feel more motivated to try new things and/or to do the things I know will help me. | 12 (14%) | 4 (3-4) | 2 (1-3) | 2 (1-4) | 2 (1-2) | 2 (1-2) | 2 (2) | |
| 12. I would like to feel less anxious about starting activities, and/or stopping them before finishing. | 9 (10%) | 4 (3-4) | 1 (1-2) | 3 (2-4)** | 2 (1-3)* | 4 (4)*** | 3 (2-4)** | |
| 13. I would like to feel less anxious about making decisions and/or planning activities. | 11 (13%) | 4 (2-4) | 1 (1) | 2 (1-2)* | 1 (1-2) | 2 (1-3)* | 2 (1-3)* | |
| 14. I would like to feel less anxious about not doing well at something, when I don’t understand things or when I make a mistake. | 20 (23%) | 4 (2-4) | 1 (1-2) | 2 (1-3)* | 1 (1-2) | 1 (1-2) | 3 (1-5)** | |
| 15. I would like to feel less anxious about meeting other people’s expectations. | 9 (10%) | 3 (2-4) | 1 (1-2) | 2 (1-4)* | 2 (1-3)* | 2 (2-3)* | 2 (1-2)* | |
| 16. I would like to understand my anxiety, my triggers and myself more. | 17 (19%) | 4 (3-4) | 1 (1-2) | 2 (1-4)* | 2 (1-3)* | 2 (2-3)* | 2 (1-3)* | |
| 17. I would like to develop some coping strategies to help with my worries and my anxiety (e.g. talking to others about my anxiety, etc). | 16 (18%) | 4 (3-4) | 1 (1-2) | 2 (1-4)* | 2 (1-2)* | 2 (1-3)* | 2 (1-5)* | |
| 18. I would like to compare myself to neurotypical people less. | 11 (13%) | 4 (3-4) | 1 (1-2) | 2 (1-5)* | 1 (1) | 1 (1-2) | 2 (1-5)* | |
| 19. I would like to worry less about things that have happened in the past. | 20 (23%) | 4 (3-4) | 1 (1-2) | 2 (1-3)* | 1 (1-2) | 1 (1-2) | 2 (1-2)* | |
| 20. I would like to feel less anxious in general. | 26 (30%) | 4 (1-4) | 1 (1-2) | 1 (1-3) | 1 (1-2) | 1 (1-2) | 1 (1-2) | |

*Calculated from total respondents for this item at baseline (T1) (N=88). *1-point change in average reported distance from achieving goal as compared to baseline (T1); **2-point change in average reported distance from achieving goal as compared to baseline (T1); ***3-point change in average reported distance from achieving goal as compared to baseline (T1).
